# ERCC6L2 mitigates replication stress and promotes centromere stability

**DOI:** 10.1016/j.celrep.2023.112329

**Published:** 2023-04-03

**Authors:** Christopher J. Carnie, Lucy Armstrong, Marek Sebesta, Antonio Ariza, Xiaomeng Wang, Emily Graham, Kang Zhu, Dragana Ahel

**Affiliations:** 1 Sir William Dunn School of Pathology, University of Oxford, Oxford OX1 3RE, UK

**Keywords:** ERCC6L2, centromere, replication stress, chromatin, DNA repair, end resection, IBMFS, PCNA, PIP-box, SNF2 ATPase

## Abstract

Structurally complex genomic regions, such as centromeres, are inherently difficult to duplicate. The mechanism behind centromere inheritance is not well understood, and one of the key questions relates to the reassembly of centromeric chromatin following DNA replication. Here, we define ERCC6L2 as a key regulator of this process. ERCC6L2 accumulates at centromeres and promotes deposition of core centromeric factors. Interestingly, *ERCC6L2*^−/−^ cells show unrestrained replication of centromeric DNA, likely caused by the erosion of centromeric chromatin. Beyond centromeres, ERCC6L2 facilitates replication at genomic repeats and non-canonical DNA structures. Notably, ERCC6L2 interacts with the DNA-clamp PCNA through an atypical peptide, presented here in a co-crystal structure. Finally, ERCC6L2 also restricts DNA end resection, acting independently of the 53BP1-REV7-Shieldin complex. We propose a mechanistic model, which reconciles seemingly distinct functions of ERCC6L2 in DNA repair and DNA replication. These findings provide a molecular context for studies linking ERCC6L2 to human disease.

## Highlights

• ERCC6L2 is a centromeric protein
• ERCC6L2^−/−^ cells show loss of centromeric factors and changes in centromeric DNA replication
• ERCC6L2 contains a PIP-box motif and facilitates replication at complex DNA structures
• ERCC6L2 acts independently of the 53BP1-REV7-Shieldin complex to restrict DNA end resection

## Graphical abstract

**Figure:**
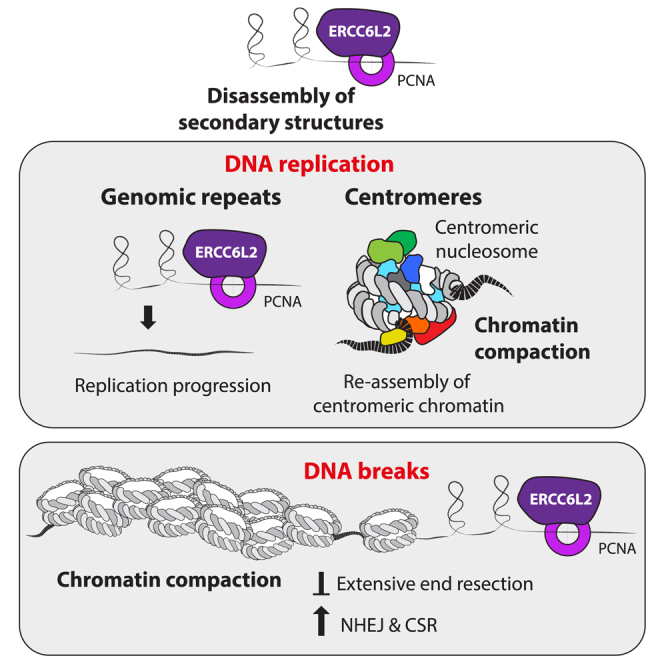

## Teaser

Carnie et al. identify ERCC6L2 as a centromeric protein, which facilitates replication of complex DNA regions and regulates the maintenance of chromatin structure. The authors offer insights into the mechanism that explains seemingly distinct functions of ERCC6L2 in DNA repair and DNA replication.

## Introduction

DNA replication is essential for the survival of proliferating cells. However, it can be perturbed by events and structures that act as replication barriers, leading to a phenomenon referred to as “replication stress.” Replication stress has important implications for human pathology and has been recognized as a driver of genomic instability during tumorigenesis.^1^^,^^2^^,^^3^^,^^4^ Important sources of replication stress include regions characterized by compact chromatin structure, DNA repeats, and sequences prone to the formation of secondary DNA structures (for example G-quadruplexes, hairpins, and cruciforms). Consequently, some genomic loci—such as common fragile sites (CFSs), telomeres, and centromeres—are particularly susceptible to replication stress.^5^^,^^6^^,^^7^^,^^8^^,^^9^

Centromeres are defined as chromosomal loci at which kinetochores—complex structures that attach to spindle microtubules—are assembled. In human chromosomes, centromeres comprise 171 base pair (bp) AT-rich repeats known as alpha-satellite DNA, which is further assembled into higher-order repeats.^10^^,^^11^ Most higher-order repeats contain a conserved 17-bp motif called the CENP-B box, which is specifically recognized by centromere protein B (CENP-B).^12^^,^^13^ However, most eukaryotic centromeres are defined epigenetically^14^ (i.e., by factors other than the DNA sequence). This is achieved by the incorporation of the histone H3 variant centromere protein A (CENP-A), which enforces conformational rigidity on the centromeric nucleosome.^15^ The compact structure of centromeric chromatin and the repetitive nature of centromeric DNA are thought to impose replication stress, and contribute to their instability.^6^ Indeed, centromeres were found to colocalize with chromosomal breakage sites,^16^ and have been associated with defective chromosomes in cancer cells.^17^^,^^18^^,^^19^ This highlights the intrinsic fragility of centromeres and raises important questions about the cellular mechanisms that ensure their integrity, which are still largely unknown despite some recent advances.^20^^,^^21^^,^^22^^,^^23^

Here we uncover the molecular links between DNA replication and functional assembly of human centromeres, and define ERCC6L2 as a key regulator of this process. ERCC6L2 (Excision Repair Cross-Complementation Group 6 Like 2), also known as RAD26L and as the HElicase mutated in Bone Marrow Failure, HEBO^24^ is a poorly characterized member of the Sucrose Non-Fermenting 2 (SNF2) family of ATPases^25^ associated with a distinct bone marrow failure syndrome.^24^^,^^26^^,^^27^^,^^28^^,^^29^^,^^30^^,^^31^ Beyond centromeres, we also consider the role of ERCC6L2 in DNA double-strand break repair, providing insights into its recently proposed roles in non-homologous end-joining (NHEJ).^32^^,^^33^^,^^34^ Our findings broaden the current perspectives on cellular strategies that mitigate replication stress, and on their possible link with human pathology.

## Results

### ERCC6L2 is a centromere-associated protein

**Figure 1:**
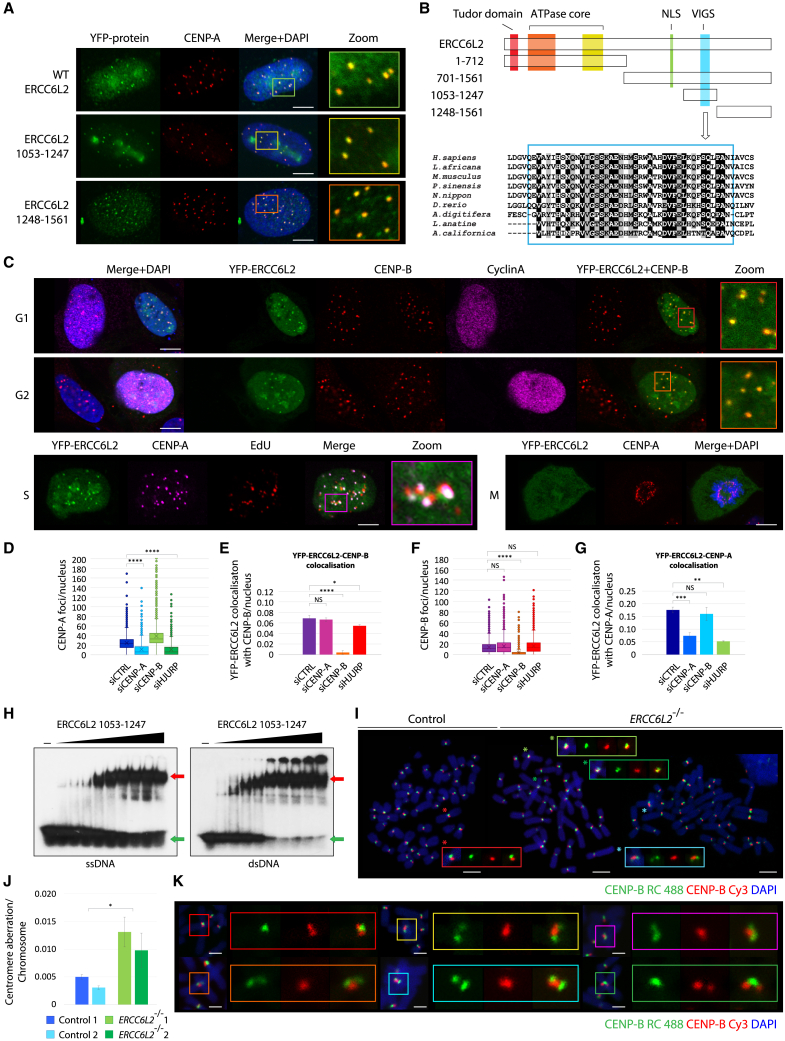
ERCC6L2 is a centromere-associated protein (A) ERCC6L2 is recruited to centromeres. YFP-ERCC6L2 constructs were expressed in U2OS cells, and then fixed and stained against the centromeric marker CENP-A. Scale bar, 5 μm. (B) Schematic representation of ERCC6L2 protein sequence. Truncated ERCC6L2 proteins used in (A), and alignment of ERCC6L2 sequences corresponding to the conserved VIGS domain, are also shown. (C) ERCC6L2 localizes to centromeres throughout interphase. YFP-ERCC6L2 was expressed in U2OS cells, which were subsequently immunostained with specified antibodies. Cyclin A immunostaining was used to distinguish G1 (Cyclin A-negative) from G2 (Cyclin A-positive) cells, while CENP-B was used as a centromeric marker. To identify S-phase cells, YFP-ERCC6L2 transfected cells were pulse labeled with EdU for 15 min and subjected to staining using Click-iT EdU Alexa Fluor 594 imaging kit. Centromeres were detected by CENP-A antibody. Mitotic cells were identified by DAPI-stained condensed chromosomes. Scale bar, 5 μm. (D) Box and whisker plot measuring the effect of the indicated small interfering RNA (siRNA) on formation of CENP-A foci. Cells were transfected with siRNA on 2 consecutive days, and with YFP-ERCC6L2 construct 10 h after first siRNA transfection. They were fixed and stained 48 h following first siRNA transfection. N(cells) > 2,200. (E) Quantification of YFP-ERCC6L2 colocalization with CENP-B in cells transfected with the indicated siRNAs. Conditions as in (D). Bars represent means with standard deviations. N(cells) > 6,000. (F) Box and whisker plot measuring the effect of the indicated siRNA on formation of CENP-B foci. Conditions as in (E). (G) Quantification of YFP-ERCC6L2 colocalization with CENP-A in cells transfected with the indicated siRNAs. Conditions as in (D). Bars represent means with standard deviations. (H) Electrophoretic mobility shift assay with the purified ERCC6L2^1053−1247^ fragment. Increasing concentrations of protein (11, 22, 44, 88, 176 nM) were incubated with radioactively labeled single-stranded (left) or double-stranded DNA (right) and resolved by native polyacrylamide gel electrophoresis. Free DNA substrates are marked by green arrows. Nucleoprotein complexes are marked by red arrows. (I) Representative images of metaphase spreads from control and *ERCC6L2*^−/−^ hTERT-RPE1 cells. Magnified views of normal and aberrant chromosomal structures are shown in colored frames. Abnormal CO-FISH patterns are marked (^∗^). Scale bar, 5 μm. (J) Frequency of aberrant centromere CO-FISH patterns in indicated cell lines. Bars represent means with standard deviations. N(chromosome) ≥ 3,600. Statistics calculated by one-way ANOVA; ^∗^p ≤ 0.05. (K) Representative images of centromere aberrations observed by CO-FISH. Magnified views of differentially labeled chromatids are shown in colored frames. Scale bar, 2 μm. (D–G) Statistics calculated by t test assuming unequal variances; ^∗^p ≤ 0.05, ^∗∗^p ≤ 0.01, ^∗∗∗^p ≤ 0.001, ^∗∗∗∗^p ≤ 0.0001, NS not significant.

We initially observed that YFP-tagged ERCC6L2 expressed in unperturbed U2OS cells forms discrete nuclear foci. We considered the possibility that these foci may correspond to specific genomic loci. Remarkably, we found that ERCC6L2 foci colocalize with CENP-A, the defining constituent of centromeric chromatin (Figures 1A and 1B). Centromeric localization of ERCC6L2 was not confined to a particular phase of the cell cycle, and could be detected throughout interphase (Figure 1C). Further analysis revealed that two fragments located at the C-terminus of the ERCC6L2 protein, YFP-ERCC6L2^1053−1247^ and YFP-ERCC6L2^1248−1561^, also formed foci marked by the CENP-A protein (Figures 1A and 1B). These observations suggested that the C-terminal region of ERCC6L2 contains the structural determinant(s) for centromeric localization.

To investigate whether core centromeric factors drive recruitment of ERCC6L2 to centromeres, we assessed YFP-ERCC6L2 localization upon downregulation of CENP-A, CENP-B, and the CENP-A chaperone HJURP. As expected, siCENP-A and siHJURP reduced the number of CENP-A foci/nucleus (Figure 1D). However, the changes in CENP-A levels did not have a significant effect on recruitment of ERCC6L2 to centromeres (assessed by the colocalization of YFP-ERCC6L2 with CENP-B; Figure 1E). Similarly, reducing the CENP-B levels by siCENP-B did not cause a consistent reduction in YFP-ERCC6L2 CENP-A colocalization (Figures 1F and 1G). Based on this, we conclude that accumulation of ERCC6L2 is not strictly dependent on the presence of CENP-A/B, possibly suggesting that the specific DNA structures, rather than the presence of centromeric protein factors, guide retention of ERCC6L2 at centromeres.

Intriguingly, fragment ERCC6L2^1053−1247^ contains a highly conserved region of unknown function (the “VIGS motif,” Figure 1B). This fragment also includes regions of positively charged amino acids, which led us to hypothesize that it might have a DNA-binding function. To test this possibility, we expressed and purified His-tagged ERCC6L2^1053−1247^ and assessed its ability to interact with DNA. As shown in Figure 1H, ERCC6L2^1053−1247^ formed stable nucleoprotein complexes with both single-stranded and double-stranded DNA.

### *ERCC6L2* deficiency results in centromere abnormalities

Our data linking ERCC6L2 to centromeres raised questions about its role in centromere integrity. To explore this, we performed chromosome-orientation fluorescent *in situ* hybridization (CO-FISH) using centromere-specific probes on wild-type and *ERCC6L2*^−/−^ hTERT-RPE1 cells. This assay allowed us to differentially label sister chromatids^35^^,^^36^^,^^37^ and discriminate between normal and aberrant centromeric CO-FISH patterns (Figure S1). Distinct mutagenic activities, including recombination, unequal sister chromatid exchange, and translocation, are believed to confer different types of centromeric abnormalities.^38^^,^^39^ We noted a specific chromosomal aberration involving two chromosomes, which appeared to be joined at their respective centromeric regions. Importantly, such chromosomes displayed abnormal CO-FISH patterns (Figures 1I, 1K, and S1), suggesting that they did not originate from stochastically overlapped chromosomes. We therefore hypothesized that they might represent centromere fusions or rearrangements, likely resulting from recombination events at α-satellite repeats located on different chromosomes (rather than at sister chromatids). Quantitative analysis revealed that these aberrations occurred more frequently in *ERCC6L2*^−/−^ cells (Figure 1J), indicating a role for ERCC6L2 in the maintenance of centromere stability.

### *ERCC6L2* deficiency causes disruption of centromeric chromatin

**Figure 2:**
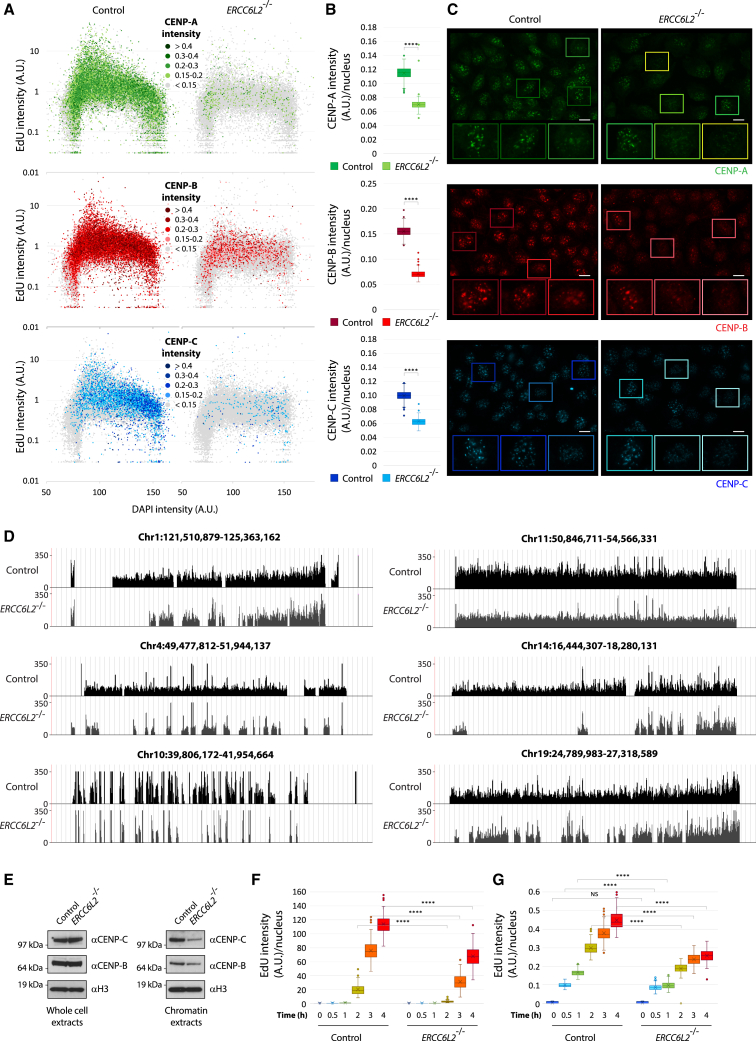
ERCC6L2 deficiency causes disruption of centromeric chromatin (A) QIBC assays measuring CENP-A, CENP-B, and CENP-C intensities in control and *ERCC6L2*^−/−^ U2OS cells. Shown is cell cycle distribution of EdU-labeled cells. Individual cells were colored according to the relative CENP-A, CENP-B, and CENP-C intensities, as indicated. N(total cells) > 30,000. (B) Box and whisker plots measuring average intensities of CENP-A, CENP-B, and CENP-C foci in control and *ERCC6L2*^−/−^ U2OS cells. N(images) > 250, N(cells) > 30,000. (C) Representative images used for quantifications in (A). Shown are expressions of centromeric proteins, as indicated, with zoomed images of cells in colored frames displaying different levels of focal intensities. Scale bar, 10 μm. (D) *ERCC6L2*^−/−^ cells show a reduced CENP-B occupancy. CENP-B ChIP-seq reads mapped to centromeric regions of the representative chromosomes. The tracks show average number of reads (obtained from triplicates in a sample group) covering a given base on the x axis. (E) ERCC6L2 deficiency does not affect expression of centromeric factors CENP-B and CENP-C. Shown are western blots of whole-cell and chromatin extracts derived from control and *ERCC6L2*^−/−^ U2OS cells. Blots against histone H3 is used as a loading control. (F) DNA replication measured as intensity of incorporated EdU during the indicated time frames in control and *ERCC6L2*^−/−^ U2OS cells. N(images) > 130, N(cells) > 20,000. (G) Quantification of DNA replication in cells exposed to 10 μM Polα inhibitor CD437 during EdU incorporation. N(images) > 130, N(cells) > 20,000. (B), (F), and (G) Box and whisker plots. Statistics calculated by t test assuming unequal variances; ^∗∗∗∗^p ≤ 0.0001, NS, not significant.

We next assessed the impact of ERCC6L2 deficiency on deposition/retention of centromeric proteins. Because centromeres are dynamically regulated throughout the cell cycle, we applied quantitative image-based cytometry (QIBC), which allowed us to identify discrete cell populations based on EdU and DAPI staining. Our initial analysis revealed that control and *ERCC6L2*^−/−^ cells displayed substantial differences in centromeric foci, despite similar cell cycle profiles (Figures 2A–2C). Specifically, average nuclear intensities of CENP-A, CENP-B, and CENP-C foci were reduced in *ERCC6L2*^−/−^ cells, suggesting that these factors might not be efficiently incorporated at centromeric loci in the absence of ERCC6L2 activity. The data also suggested that the observed differences in centromeric intensities were not confined to a particular stage of the cell cycle. To further explore this, we performed QIBC using Cyclin A and DAPI staining, which allowed us to discriminate between the specific stages of the cell cycle more precisely (Figure S2A). We found that CENP-B and CENP-C intensities gradually increased with the cell cycle progression, suggesting that centromeres are reassembled on newly replicated DNA. While this trend was observed in both cellular backgrounds, *ERCC6L2*^−/−^ cells showed significant reductions in average CENP-B and CENP-C intensities throughout the cell cycle (Figures S2B and S2C).

Our QIBC data suggested a role of ERCC6L2 in the deposition of centromeric factors following DNA replication. To further validate this, we identified precise genomic locations of CENP-B binding sites using chromatin immunoprecipitation sequencing (ChIP-seq) analysis. This allowed us to compare genomic maps of CENP-B distribution in control and *ERCC6L2*^−/−^ backgrounds (Figure 2D). As expected, identified CENP-B peaks were clustered at centromeric sites in both cell lines. However, comparative analysis revealed a substantial decrease in CENP-B coverage in *ERCC6L2*^−/−^ cells, in agreement with our previous observations. Importantly, disruptions in centromeric chromatin in *ERCC6L2*^−/−^ cells were not caused by changes in the proteins’ expression levels (Figure 2E). Collectively, these results suggested a role of ERCC6L2 in the proficient assembly of functional centromeres.

### ERCC6L2 loss perturbs replication dynamics

Because centromeres impose specific challenges for DNA replication machinery, we next sought to address the impact of ERCC6L2 deficiency on the progression of DNA replication. Molecular combing, applied to measure incorporation of the nucleoside analogues CldU and IdU during a short pulse (40 min), revealed similar replication fork speeds in control and *ERCC6L2*^−/−^ cells (Figures S2D and S2E). However, further analyses, based on EdU incorporation over longer time frames, highlighted differences between the two cell lines. Loss of *ERCC6L2* reduced efficiency of EdU incorporation in unperturbed conditions (Figure 2F). Similar observations were made when cells were exposed to the Polα inhibitor CD437, which causes uncoupling of leading and lagging strand DNA synthesis (Figure 2G).^40^ These findings suggest that ERCC6L2 promotes the timely progression of DNA replication, possibly through localized effects at specific loci.

### Genome-wide analysis reveals differences in centromeric replication

**Figure 3:**
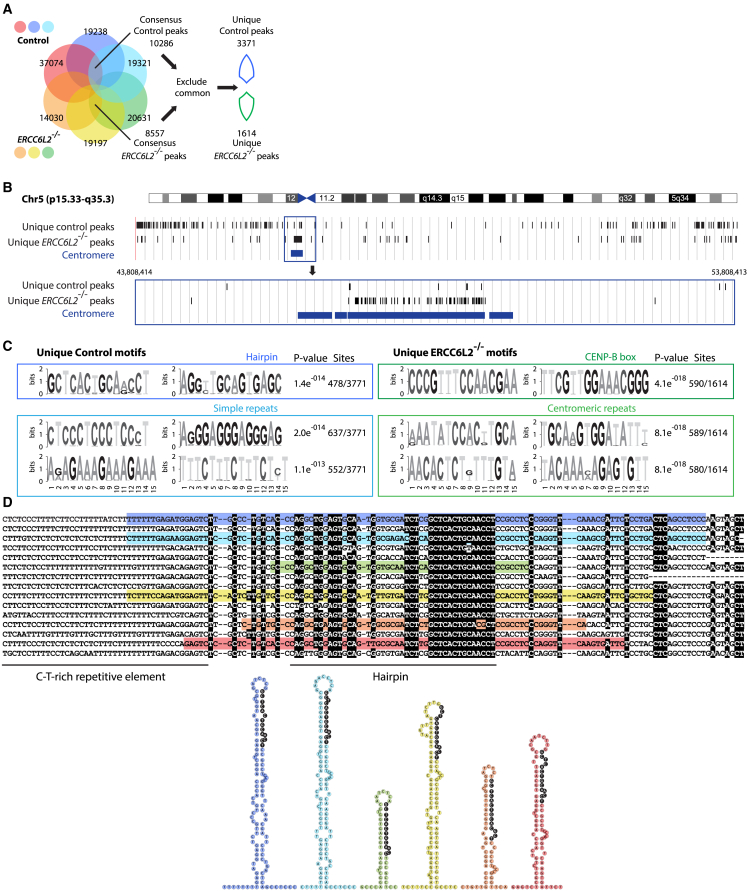
ERCC6L2 regulates centromeric chromatin and alleviates replication stress at genomic repeats (A) Non-proportional Venn diagram illustrating the overlap between the peaks in control and *ERCC6L2*^−/−^ triplicates identified by nascent DNA sequencing in U2OS cells. The experiment provides a snapshot of replicated DNA over the duration of BrdU pulse in an asynchronous cell population, and is expected to include regions replicated at different stages of S-phase. Direct comparison between control and *ERCC6L2*^−/−^ peaks identifies regions that are, in relative terms, under- or over-replicated in specific genetic backgrounds. Shown are consensus control and *ERCC6L2*^−/−^ peaks (common to each genetic background), and unique control and *ERCC6L2*^−/−^ peaks (exclusive to each genetic background). (B) Distribution of unique control and *ERCC6L2*^−/−^ peaks on chromosome 5 identified by nascent DNA sequencing. Zoomed view of the framed 10-Mb region is shown below. (C) Top recurring motifs among unique control and *ERCC6L2*^−/−^ peaks identified by the MEME suite.^41^ Shown are p values and numbers of sites. (D) Conservation of the regions surrounding the top recurring motif identified among unique control peaks (Figure 3E). Alignment reveals presence of the C-T rich repetitive element upstream of the conserved motif. Secondary structures of selected sequences, predicted by RNA fold server, are shown below. The conserved motif is predicted to constitute a part of a hairpin structure.

While our results suggested a role for ERCC6L2 in proficient DNA replication, the precise genomic loci suffering compromised replication in the absence of ERCC6L2 remained unclear. To address this, we compared DNA replication in control and *ERCC6L2*^−/−^ U2OS cells by nascent DNA sequencing. Specifically, we pulsed cells with the nucleoside analogue BrdU and isolated labeled DNA by anti-BrdU immunoprecipitation. Purified nascent DNA was then analyzed by next-generation sequencing (NGS). We identified peaks consistently represented within each of the triplicate groups (“consensus peaks”), which we then compared to define “unique peaks,” conserved only within the specified genetic background (Figure 3A). This allowed us to define genomic regions that were replicated only in the presence, or only in the absence of ERCC6L2.

Direct comparison of the unique peaks revealed differences in replication patterns between control and *ERCC6L2*^−/−^ cells. Unlike unique control peaks, which were more evenly distributed through chromosome arms, unique *ERCC6L2*^−/−^ peaks appeared in concentrated clusters. Strikingly, these clusters of unique *ERCC6L2*^−/−^ peaks directly overlapped with alpha-satellite centromeric regions (Figures 3B and S3A). This suggested that replication of centromeric DNA is less constrained in the absence of ERCC6L2 protein, likely due to the reduced occupancy of centromeric proteins in *ERCC6L2*^−/−^ cells (Figures 2A–2D). Based on this, we conclude that, in ERCC6L2 proficient setting, chromatin compaction acts as the dominant force in imposing replication slowdown at centromeres.

To gain further insight, we searched unique peaks for the presence of recurring motifs. Remarkably, the top hit among unique *ERCC6L2*^−/−^ peaks matched the CENP-B binding site (CENP-B box; Figure 3C). The CENP-B box was present in more than 35% unique *ERCC6L2*^−/−^ peaks. Moreover, all of the identified top 10 *ERCC6L2*^−/−^ hits corresponded to centromeric sequences (Figure S3B). In contrast, none of the top recurring motifs among unique control peaks mapped to centromeric loci. Instead, identified control hits contained repetitive elements (including CCCT/AGGG, AAAG/CTTT, AAGG/CCTT, and AGA/TCT repeats; Figures 3C and S3B). We further inspected the top unique control motif, and found that it constituted a part of a conserved region, characterized by a C-T-rich repetitive element and a sequence predicted to form a stable hairpin structure (Figure 3D). Importantly, these sequences were not found among unique *ERCC6L2*^−/−^ peaks.

Collectively, our data suggest that ERCC6L2 facilitates replication through repetitive genomic regions and non-canonical DNA structures. Our results also indicate that replication of centromeric DNA faces fewer obstacles in *ERCC6L2*^−/−^ cells, characterized by the disruption of centromeric chromatin (Figures S3A and 2A–2D). These observations highlight contributions of both DNA repeats and repressive chromatin structure in imposing replication stress, and define a central role of ERCC6L2 in this context.

### ERCC6L2 contains an atypical PCNA-binding motif

**Figure 4:**
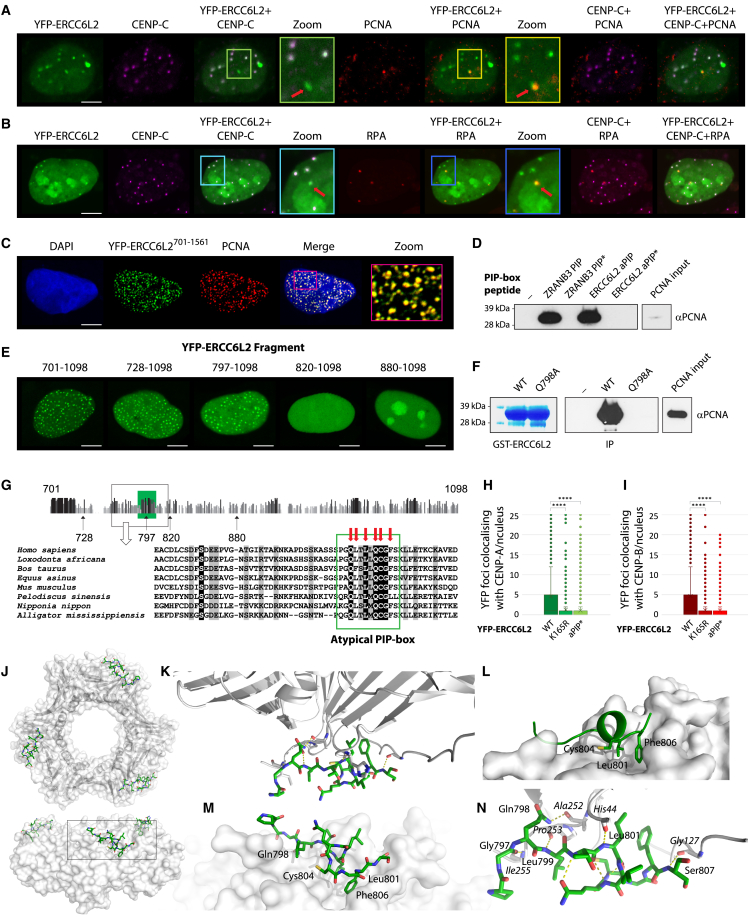
ERCC6L2 contains an atypical PCNA-binding motif (A) ERCC6L2 colocalizes with PCNA at CENP-C-negative foci. U2OS cells transfected with YFP-ERCC6L2 were stained for CENP-C and PCNA after pre-extraction of soluble proteins. ERCC6L2-and PCNA-positive, but CENP-C negative foci (red arrows) are marked in zoomed images. (B) ERCC6L2 colocalizes with RPA at CENP-C-negative foci. U2OS cells transfected with YFP-ERCC6L2 were stained for CENP-C and RPA. ERCC6L2- and RPA-positive, but CENP-C-negative foci (red arrows) are marked in zoomed images. (C) Colocalization of the C-terminal ERCC6L2^701−1561^ fragment with endogenous PCNA. (D) PCNA pull-down with biotinylated peptides. Biotinylated PIP-box and APIM motif peptides were bound to streptavidin beads and incubated with recombinant PCNA. Interactions were assayed by western blotting with PCNA antibody. Wild-type ZRANB3 PIP-box peptide (ZRANB3 PIP) was used as a positive control. Mutant ZRANB3 PIP-box peptide (Q519A, F525A, and F526A, ZRANB3 PIP^∗^) served as a negative control. ERCC6L2 aPIP and ERCC6L2 aPIP^∗^ denote atypical ERCC6L2 peptides (residues 790–811) containing the WT and the Q798A mutant sequences, respectively. (E) Expression patterns of the indicated C-terminal YFP-ERCC6L2 constructs. ERCC6L2 fragments 701–1,098, 728–1,098, and 797–1,098 show patterns reminiscent of replication foci, whereas fragments 820–1,098 and 880–1,098 do not. (F) PCNA pull-down with GST-tagged peptides. Wild-type and Q798A mutant versions of the GST-tagged ERCC6L2 fragments (residues 753–819) were immobilized on GST beads (shown on the left) and incubated with recombinant PCNA. Interactions were assessed by western blotting against PCNA (shown on the right). (G) Schematic representation of different ERCC6L2 fragments tested for formation of PCNA-like foci. A region at the N terminus of fragment 701–1,098 is zoomed below to show sequence alignment of different ERCC6L2 proteins. The conserved atypical PIP-box is marked in a green frame. Targeted mutation sites are indicated by red arrows. (H) Colocalization of YFP-ERCC6L2 wild type, ATPase dead K165 ERCC6L2, and aPIP^∗^ mutant (Q798A, C804A, F806A) with CENP-A. N(cells) > 1,400. (I) Colocalization of YFP-ERCC6L2 wild type, ATPase dead K165 ERCC6L2, and aPIP^∗^ mutant (Q798A, C804A, F806A) with CENP-B. N(cells) > 1,000. (J) Front and side views of the PCNA ring (white surface and ribbons) with the ERCC6L2 aPIP-box peptide (green sticks). (K) Overview of the hydrogen-bond interaction network between the ERCC6L2 aPIP-box peptide (green) and PCNA (white). Hydrogen bonds are shown as yellow dotted lines. (L) Hydrophobic pocket on PCNA surface (white) with conserved residues that form “hydrophobic plug” (Leu801, Cys804, and Phe806; shown as green sticks) in the ERCC6L2 aPIP-box peptide. (M) Magnified view of the boxed region in (I). (N) Magnified view of the hydrogen-bond interaction network between the ERCC6L2 aPIP-box peptide (green) and PCNA (white, labels in italics). Hydrogen bonds are shown as yellow dotted lines. Intramolecular hydrogen bonds between ERCC6L2 aPIP-box residues are also shown. (H) and (I) Box and whisker plots. Statistics calculated by t test assuming unequal variances; ^∗∗∗∗^p ≤ 0.0001. (A), (B), (C), (E) Scale bar, 5 μm.

Our data linking ERCC6L2 to DNA replication prompted us to search for possible structural determinants underlying this function. Interestingly, although most YFP-ERCC6L2 foci colocalized with centromeric markers (for example, CENP-C), we also observed the absence of CENP-C signal at a subset of YFP-ERCC6L2 foci. Intriguingly, these CENP-C-negative YFP-ERCC6L2 foci colocalized with the replication-associated markers, PCNA and RPA (Figures 4A and 4B). Further analyses showed that although full-length ERCC6L2 was largely absent from PCNA foci in S-phase cells, two C-terminal ERCC6L2 fragments (ERCC6L2^701−1561^ and ERCC6L2^701−1247^, Figure S4A) efficiently colocalized with endogenous PCNA (Figures 4C and S4B). Endogenous PCNA was also detected in ERCC6L2^701−1053^ immunocomplexes after immunoprecipitation (Figure S4C).

Interactions with PCNA are typically mediated through PCNA-interaction motifs, such as the PIP-box (Q-x-x-[VILM]-x-x-[FY]-[FY]) and the APIM motif ([KR]-[FYW]-[LIVA]-[LIVA]-[KR])^42^^,^^43^; however, these motifs could not be identified in the primary sequence of ERCC6L2^701−1053^. Therefore, to define the region of the ERCC6L2 protein that interacts with PCNA, we generated a series of truncations of the ERCC6L2^701−1098^ fragment. The resulting constructs were expressed as YFP-tagged proteins and tested for the presence of nuclear patterns resembling replication foci. Our analysis suggested that the ERCC6L2 region between residues 797 and 820 contains a putative PCNA-binding motif (Figure 4E).

To test this possibility, we examined the primary sequence and noted a conserved stretch of amino acids defined by the Q-[FL]-x-L-x-Q-C-G-[FL] consensus (Figure 4G). While showing some similarities to the PIP-box, this motif deviates from the canonical PIP-box sequence. We therefore proceeded to validate its ability to mediate interactions with PCNA. The interaction with PCNA could be demonstrated by using immobilized GST-tagged ERCC6L2^753−819^ protein, or biotinylated ERCC6L2^790−811^ peptide (henceforth referred to as atypical or aPIP-box) (Figures 4D and 4F). The interaction of ERCC6L2 aPIP-box with PCNA was comparable to the one observed with the classical PIP-box peptide from ZRANB3^44^^,^^45^ (Figure 4D). Isothermal titration calorimetry (ITC) revealed a K_D_ value of approximately 6 μM, which was equivalent to the values detected for other PIP-box and APIM peptides (Figure S4D^45^). Further experiments confirmed the relevance of the conserved aPIP-box residues for PCNA binding (Figures S4E and S4F).

Importantly, mutation of the aPIP-box compromised the ability of ERCC6L2 to colocalize with centromeric factors CENP-A and CENP-B, as well as with sites of DNA replication (YFP-ERCC6L2 aPIP^∗^, Figures 4H, 4I, S4G, and S4H). Similar results were obtained upon the mutation of the ERCC6L2 ATPase active site (YFP-ERCC6L2 K165R, Figures 4H, 4I, S4G, and S4H). These results suggest that proficient accumulation/retention of ERCC6L2 at specific sites involves its active translocation via a stable, PCNA-bound complex.

### Structural insights into the ERCC6L2 aPIP:PCNA interaction

Since the ERCC6L2 aPIP-box peptide diverges from the canonical PIP-box sequence, we crystallized and determined its co-structure with PCNA (Table S1). The complex revealed a typical homo-trimeric association of PCNA monomers, each with its own ERCC6L2 aPIP-box peptide (Figures 4J and S4I). The ERCC6L2 aPIP-box peptide displayed some classical features observed with other PCNA-binding peptides. It formed a 3_10_ helical turn (Figures 4K and 4L), constraining Leu801, Cys804, and Phe806 into a trident structure known as the “hydrophobic plug.” This was critical for the efficient docking of the peptide into the hydrophobic pocket on the PCNA surface (Figures 4L and 4M). Binding to PCNA was additionally supported by a network of electrostatic interactions, with the conserved glutamine residue Gln798 playing a key role (Figure 4N).

Despite the similarities between the ERCC6L2 aPIP-box and other PCNA-binding peptides in the basic configuration and the mode of binding, the ERCC6L2 aPIP-box displayed two major idiosyncrasies. First, spacing of the hydrophobic residues in the ERCC6L2 aPIP-box peptide did not conform to the conventional consensus Φ_1_-x-x-Φ_2_-Φ_3_. Instead, it included an additional residue and was identified by the Φ_1_-x-x-Φ_2_-x-Φ_3_ sequence. Second, a cysteine residue (Cys804) occupied one of the defining positions of the hydrophobic plug, which is unprecedented. These differences suggest that PCNA-binding peptides are more versatile than currently appreciated.

### Role of ERCC6L2 in DNA double-strand break end resection

ERCC6L2 has recently been implicated in NHEJ,^32^^,^^33^^,^^34^ but the exact molecular mechanism of its action remains unclear. We initially observed that *ERCC6L2* deficiency resulted in increased sensitivity to the DNA double-strand break (DSB)-inducing agents, the topoisomerase II inhibitor etoposide, and to the radiomimetic phleomycin (Figure S5A^32^). We also observed efficient mobilization of YFP-tagged ERCC6L2 to laser-induced DNA breaks, supported by the C-terminal region of the protein (Figure S5B).

**Figure 5:**
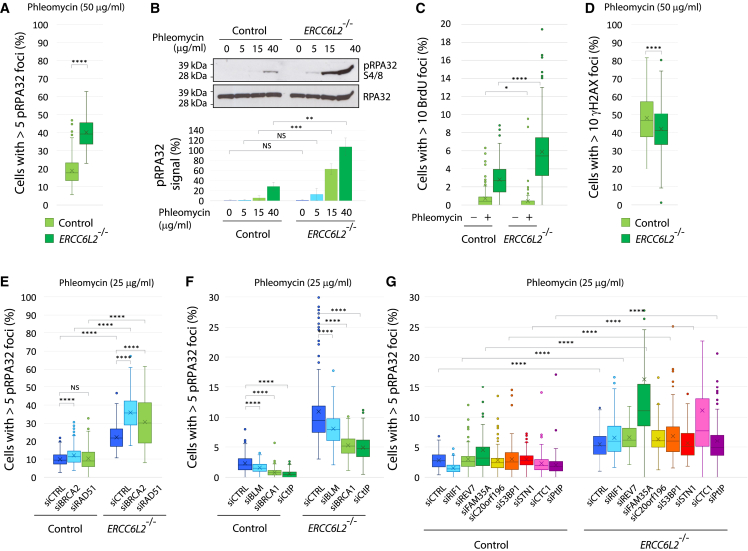
ERCC6L2 deficiency causes DSB hyper-resection (A) Quantification of ssDNA accumulation using phosphorylated RPA32 (pRPA) as a marker in control and *ERCC6L2*^−/−^ U2OS cells. Cells were treated with 50 μg/mL phleomycin for 1 h, and allowed to recover in normal media for 6 h. N(images) > 250, N(cells) > 30,000. (B) Quantification of pRPA levels by western blot. Cells were treated overnight with the indicated doses of phleomycin. pRPA signal, quantified by ImageJ, is expressed as % of total RPA signal, and calculated as an average of three experiments. (C) Quantification of BrdU-labeled ssDNA. Cells were labeled with BrdU for 24 h, treated with 50 μg/mL phleomycin for 1 h, and allowed to recover for 6 h. BrdU immunostaining was performed under native conditions. (D) Quantification of γH2AX foci in cells treated with 50 μg/mL phleomycin for 1 h and allowed to recover in normal media for 6 h. (E) Downregulation of BRCA2 or RAD51 exacerbates pRPA accumulation in and *ERCC6L2*^−/−^ cells. Cells were transfected with the indicated siRNAs, treated with 25 μg/mL phleomycin for 1 h, and allowed to recover in normal media for 6 h. (F) Quantification of pRPA foci in control and *ERCC6L2*^−/−^ U2OS cells transfected with the indicated siRNAs. Cell treatments were performed as in (E). (G) Downregulation of 53BP1 effector proteins exacerbates pRPA accumulation in *ERCC6L2*^−/−^ cells. Cells were transfected with the indicated siRNAs, and treated as in (E). N(images) > 250, N(cells) > 30,000. (A–G) Box and whisker plots. Statistics calculated by t test assuming unequal variances; ^∗^p ≤ 0.05, ^∗∗^p ≤ 0.01, ^∗∗∗^p ≤ 0.001, ^∗∗∗∗^p ≤ 0.0001, NS, not significant.

These observations prompted us to examine whether ERCC6L2 affects critical steps in DSB detection and processing. To assess its impact on end resection, we used phosphorylated RPA32 (pRPA) as a marker of single-stranded DNA (ssDNA) generated at resected DSBs.^46^ Interestingly, we found that the loss of *ERCC6L2* resulted in increased levels of pRPA following induction of DSBs (Figures 5A, 5B, and S5C). Direct visualization of BrdU-labeled ssDNA using native BrdU staining showed a similar trend (Figure 5C). The kinetics of the pRPA accumulation indicated a progressive accumulation of ssDNA in *ERCC6L2*^−/−^ cells, with more notable differences at later time points (Figure S5D). Importantly, we observed elevated pRPA, but not γH2AX foci in *ERCC6L2*^−/−^ cells (Figure 5D). This suggested that *ERCC6L2*^−/−^ cells did not incur higher levels of DNA damage, and that the observed increase in pRPA was related to DSB processing.

DSB end processing dictates the choice between the two principal pathways of DSB repair.^47^ NHEJ only acts on minimally processed DNA ends, while homologous recombination (HR) requires extensive end resection. Since RPA is replaced by RAD51 at resected DNA ends in the subsequent steps of HR, we next disrupted RAD51 nucleofilament formation by RAD51 or BRCA2 depletion. This caused an increase in the damage-induced pRPA levels, which were more pronounced in *ERCC6L2*^−/−^ cells (Figures 5E and S5E). In contrast, levels of pRPA foci were reduced by depleting factors that promote or modulate end resection, such as CtIP (RBBP8), BLM, and BRCA1^48^ (Figure 5F).

We also examined a possible link with 53BP1 and its downstream effectors tied to the control of DSB repair pathway choice. 53BP1 protects DSBs against hyper-resection through interactions with its partner proteins, including PtIP, RIF1, REV7, and components of the Shieldin (C20orf196 [SHLD1]-FAM35A [SHLD2]-FLJ26957 [SHLD3]) and CST (CTC-STN1-TEN1) complex.^49^^,^^50^^,^^51^^,^^52^^,^^53^^,^^54^^,^^55^^,^^56^^,^^57^^,^^58^^,^^59^^,^^60^^,^^61^^,^^62^^,^^63^^,^^64^^,^^65^ We found that downregulation of FAM35A and CTC1 led to a significant increase in pRPA foci in *ERCC6L2*^−/−^ cells (Figures 5G, S5F, and S5G), suggesting a lack of simple epistasis. Interestingly, FAM35A and CTC1 both contain ssDNA-binding OB folds, analogous to OB folds in RPA. Hyper-resection in *ERCC6L2*^−/−^ cells might therefore foster competition between RPA and FAM35A/STN1 for the mutual ssDNA substrate.

Last, we explored a possible role of ERCC6L2 in protecting nascent DNA at stalled replication forks. Replication forks are known to reverse in response to replication stress, thereby generating a DNA end that is susceptible to nucleolytic degradation.^66^^,^^67^ As such ends resemble DSBs, protection of nascent DNA ends is known to involve activities of DSB repair factors, including 53BP1 and RIF1.^68^ To assess a possible involvement of ERCC6L2 in fork protection, we sequentially incubated the cells with CldU and IdU analogues, and exposed them to hydroxyurea to induce nascent DNA degradation. However, we did not observe significant differences in nascent DNA protection between control and *ERCC6L2*^−/−^ cells in these conditions (Figure S5H). Possibly, the observed differences in the end protection activities of ERCC6L2 reflect differences in the chromatin contexts at reversed forks and phleomycin-induced DSBs.

### *ERCC6L2* deficiency is associated with nuclear abnormalities

**Figure 6:**
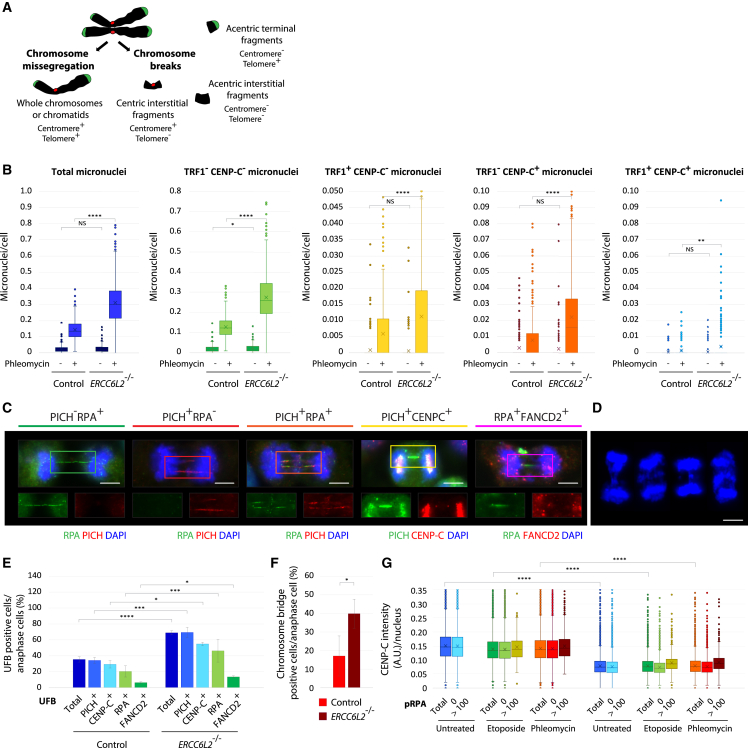
ERCC6L2 deficiency is associated with nuclear abnormalities (A) Categorization of micronuclei based on the presence of centromeric and telomeric signals. Micronuclei can arise as a consequence of chromosome missegregation (centromere- and telomere-positive micronuclei) or chromosome breaks (centromere-positive telomere-negative, centromere-negative telomere-positive, and centromere- and telomere-negative micronuclei). Interstitial fragments (centric, i.e., centromere-positive, or acentric, i.e., centromere-negative) are generated by two DSBs. Terminal fragments are telomere-positive and can be formed by a single DSB. (B) Box and whisker plots measuring micronuclei subtypes in control and *ERCC6L2*^−/−^ U2OS cells, as indicated. N(images) > 400, N(cells) > 30,000. (C) Representative images of UFBs stained with PICH, RPA, CENP-C, and FANCD2 antibodies. Cells were exposed to 25 μg/mL phleomycin for 1 h and recovered in VE-821 for 2 days. Scale bar, 5 μm. (D) Representative images of chromosome segregation defects observed in DAPI-stained *ERCC6L2*^−/−^ cells. Cell treatments were as in (C). Scale bar, 5 μm. (E) Quantification of UFB subtypes in control and *ERCC6L2*^−/−^ U2OS cells. Cell treatments were as in (C). Bars represent means with standard deviations. N(total anaphase cells) > 200. (F) Quantification of chromosome segregation defects in control and *ERCC6L2*^−/−^ U2OS cells. Cell treatments were as in (C). Bars represent means with standard deviations. N(total anaphase cells) > 200. (G) CENP-C intensities are not affected by DNA hyper-resection. To minimize the effect of the cell cycle on centromere measurements, cells were arrested in G2 by a 12-h exposure to CDK1 inhibitor RO3306. Shown is the box and whisker plot measuring CENP-C intensities in different subpopulations (in total cells, cells without and with >100 pRPA foci). N(cells) > 50,000. (B), (E–G) Statistics calculated by t test assuming unequal variances; ^∗^p ≤ 0.05, ^∗∗^p ≤ 0.01, ^∗∗∗^p ≤ 0.001, ^∗∗∗∗^p ≤ 0.0001, NS, not significant.

In line with the role in DSB repair, ERCC6L2 deficiency also caused an increased frequency of micronuclei formation following exposure to phleomycin or ATR inhibitor VE-821 (Figures 6A, 6B, and S6A). Micronuclei are defined as fragments of chromosomal material that are not incorporated into daughter cell nuclei during cell division.^69^ The presence of centromeric and telomeric signals can be used to categorize micronuclei according to their origin (Figure 6A).^70^ We found a significant increase in both telomere-positive (TRF1^+^) and centromere-positive (CENP-C^+^) micronuclei in *ERCC6L2*^−/−^ cells, compared with the control cell line (Figure 6B). Although most micronuclei originated from chromosome breaks (TRF1^+^CENP-C^−^, TRF1^−^CENP-C^+^, and TRF^−^CENP-C^−^ micronuclei; Figure 6A), TRF1^−^CENP-C^+^ levels were more specifically affected by ERCC6L2 deficiency. Additional analyses showed an increase in 53BP1 or γH2AX-positive micronuclei in *ERCC6L2*^−/−^ cells, and an increase in centromere- and telomere-positive micronuclei containing 53BP1 or γH2AX foci (Figure S6B). Collectively, these results suggest that damage-induced chromosome breaks occur more frequently in the absence of ERCC6L2.

We also analyzed formation of ultrafine anaphase bridges (UFBs), delicate DNA structures that link separating sister chromatids during anaphase. UFBs are known to arise from under-replicated DNA at centromeres, CFSs and telomeres,^71^ but also from unresolved recombination intermediates.^72^ Distinct stages of UFB formation and disassembly, as well as different types of UFBs, can be discriminated by the presence of specific marker proteins, such as PICH, RPA, FANCD2, and centromeric and telomeric proteins. Comparisons of the control and *ERCC6L2*^−/−^ cells revealed an increase in UFBs in *ERCC6L2*^−/−^ cells exposed to genotoxic stress (Figures 6C, 6E, and S6C). Further analysis showed that different types of UFBs were increased in *ERCC6L2*^−/−^ background (Figure 6E).

Interestingly, our data also suggested that at least some micronuclei generated in *ERCC6L2*^−/−^ cells result from chromosome missegregation (TRF^+^CENP-C^+^ micronuclei, Figures 6A and 6B). Analysis of anaphase cells supported this observation, showing increased frequency of chromosome bridges in treated *ERCC6L2*^−/−^ cells (Figures 6D, 6F, S6D, and S6E).

### ERCC6L2 functions at centromeres independently of DNA damage

Given our data implicating ERCC6L2 in both centromere stability, and regulation of DSB repair, we sought to assess the impact of DNA damage on centromeric markers. Interestingly, CENP-B intensities were largely unaffected by genotoxic stress, in both control and *ERCC6L2*^−/−^ cells (Figure S7A). Instead, CENP-B levels followed cell cycle dynamics, and were consistently reduced in the ERCC6L2-deficient background. To directly assess the impact of end resection on centromeres in these conditions, we identified distinct cell populations based on the levels of pRPA marker. Our analysis showed that CENP-C levels were unaffected by end resection, but were instead decreased in the absence of functional ERCC6L2 (Figure 6G). These observations led us to conclude that ERCC6L2 functions at centromeres independently of DNA damage.

Our results suggest that centromere integrity is contingent on the presence of both centromeric factors and ERCC6L2 activity. We therefore wanted to understand the relationship between their individual contributions in promoting centromere stability. To explore this, we measured micronuclei formation upon depletion of specific centromeric proteins. Our results showed that downregulation of CENP-B caused an increase in both total and centromere-positive micronuclei (Figures S7B and S7C); however, micronucleation induced by CENP-B depletion was more pronounced in the absence of ERCC6L2 activity. The additive effect between ERCC6L2 and CENP-B deficiencies suggested a lack of epistasis between these factors in promoting nuclear and centromeric integrity.

### *ERCC6L2*^−/−^ cells show signs of altered chromatin structure

Chromatin structure has been identified as one of the main mechanisms that controls end resection.^48^^,^^73^ To assess the general state of chromatin that may be contributing to the hyper-resection phenotype in *ERCC6L2*^−/−^ cells, we compared different chromatin marks in control and *ERCC6L2*^−/−^ cells. We noted a significant reduction in H3K27me3, H3K27me2, and H1 levels (Figures S8, S9 and S11), but not in the levels of many other chromatin factors (Figures S10 and S11). Considering the roles of H1 and H3K27me3 in chromatin compaction and facultative heterochromatin, the observed differences indicate changes in the higher-order chromatin structure of *ERCC6L2*^−/−^ cells.

To further validate these observations, we measured damage-induced phosphorylation of KAP1 (pKAP1) in control and *ERCC6L2*^−/−^ cells. KAP1 (TRIM28) is a known ATM substrate, phosphorylated in response to DNA damage at heterochromatic loci.^74^^,^^75^^,^^76^ We detected both decreased levels of pKAP1, and increased levels of pRPA, in phleomycin-treated *ERCC6L2*^−/−^ cells (Figures S12A and S12F). Interestingly, *ERCC6L2*^−/−^ cells also showed reduced numbers of KAP1 foci (Figures S12G and S12H), possibly due to inefficient recruitment of KAP1 to heterochromatic loci. Collectively, these results are consistent with our previous observations, and suggest alterations in heterochromatic landscape that might affect the control of end resection in *ERCC6L2*^−/−^ cells.

### Pathological *ERCC6L2* variants display functional deficiencies

Mounting evidence links ERCC6L2 to a distinct inherited bone marrow failure syndrome (IBMFS) that includes developmental delay, microcephaly, and predisposition to cancer.^24^^,^^26^^,^^27^^,^^28^^,^^29^^,^^30^^,^^31^ IBMFSs are human conditions that affect one or several cell lineages of the hemopoietic system. Other clinical and hematological complications have been associated with ERCC6L2 deficiency, including myelodysplastic syndrome (MDS) and acute myeloid leukemia (AML)^26^^,^^31^; however, the exact molecular mechanism underlying the pathology of *ERCC6L2*-associated syndrome is not well understood.

Our insight into the molecular function of ERCC6L2 allowed us to interpret the functional significance of IBMFS *ERCC6L2* variants. Analysis of the IBMFS-associated mutations revealed that they are not concentrated within any specific functional domain (Figure S13A). Two homozygous missense mutations (D283N and S669N) targeted conserved residues in the N-terminal ATPase core. Notably, Asp283 is a defining residue of the ATP-binding fold (Walker B motif), and its mutation is expected to result in the loss of ATPase activity.

Interestingly, the vast majority of variants were predicted to yield truncated products (Figure S13B), suggesting significant functional defects. Indeed, IBMFS-associated mutations compromised ERCC6L2’s recruitment to centromeres, and to sites of laser-induced DNA damage (Figures S13B and S13C). Collectively, our data suggest a high incidence of loss-of-function mutations in ERCC6L2 that affect its nuclear functions and lead to pathological outcomes.

## Discussion

Our results define a central role for ERCC6L2 in the maintenance of centromere stability. We show that ERCC6L2 accumulates at centromeric loci, and that this is facilitated by its interaction with PCNA, mediated via an atypical aPIP-box (Figures 4H–4N). Accumulation of ERCC6L2 at specific loci also requires an active ATPase motor (Figures 4H, 4I, S4G, S4H, and S7D), suggesting that translocase activity underpins this process. In this sense, PCNA might serve as a platform that provides processivity, similar to its established role in DNA replication.

**Figure 7:**
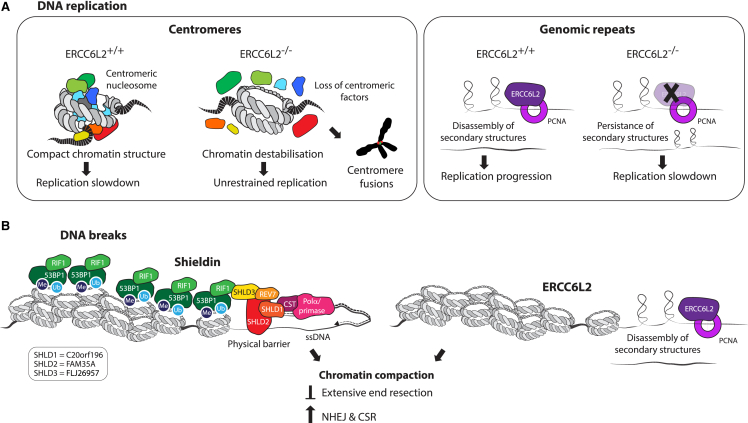
Roles of ERCC6L2 in DNA replication and at DNA breaks (A) Proposed model of ERCC6L2 role in DNA replication. ERCC6L2 counteracts replication stress at centromeric chromatin (left) and genomic repeats (right). Left: compact chromatin structure at centromeres of *ERCC6L2*^+/+^ cells causes replication slowdown. In contrast, loss of centromeric proteins from α-satellite repeats in *ERCC6L2*^−/−^ cells removes the critical epigenetic mark that underlies biological definition of centromeres, ultimately causing “centromere erosion.” Resulting deprotected centromeric DNA is replicated more rapidly, but is not properly assembled into centromeric chromatin. Potentially, deprotected centromeric DNA is also more accessible for operations with non-physiological outcomes, such as centromere fusions. Right: secondary structures that form at genomic repeats impose replication stress, which is alleviated by ERCC6L2 activity in *ERCC6L2*^+/+^ cells. In the absence of ERCC6L2, secondary structures persist and cause replication slowdown. (B) Proposed model of ERCC6L2 role at DNA breaks. In ERCC6L2-proficient cells 53BP1-REV7-Shieldin-CST complex and ERCC6L2 act independently to restrict excessive DNA end resection, and potentiate NHEJ and CSR.

Mechanistically, we propose that ERCC6L2 alleviates replication stress, possibly by using a loop-extrusion activity characteristic for this group of SNF2 ATPases.^77^ This is supported by our NGS data, which show that efficient replication of repetitive elements and hairpin forming sequences necessitates ERCC6L2 proficiency (Figures 3C, 3D, and S3B). Interestingly, centromeric DNA replication was not repressed, but dramatically enhanced, in the absence of ERCC6L2. This might seem counterintuitive, considering the repetitive nature of centromeric sequences. However, centromeres are epigenetically defined by compact chromatin structure, which in normal cells enforced replication slowdown (Figure 7A). Consequently, loss of this restrictive barrier observed in *ERCC6L2*^−/−^ cells (Figures 2A–2D) resulted in enhanced replication of centromeric repeats (Figure S3A).

ERCC6L2 could also have a role in remodeling of centromeric chromatin. Erosion of centromeric factors from *ERCC6L2*^−/−^ chromatin might suggest that their deposition following DNA replication is impaired in the absence of ERCC6L2 activity. Interestingly, available structural data show that binding of CENP-B to the CENP-box requires DNA bending by ∼60°.^78^ Consequently, centromeric repeats might be refractory to such bending and assembly of centromeric chromatin in the absence of ERCC6L2. Ultimately, loss of centromeric chromatin is expected to cause “deprotection” of centromeric DNA. As a result, such DNA might be more amenable to activities that require direct DNA contacts, and are otherwise suppressed by chromatin compaction. The increase in a specific class of centromeric aberrations observed in *ERCC6L2*^−/−^ cells (Figures 1I–1K), which seemed to originate from recombination events, appears to support this notion.

Considering that ERCC6L2 also counteracts DSB end resection, its role in conferring chromatin compaction might extend beyond centromeres (Figure 7B). Indeed, our results suggest that heterochromatin assembly and chromatin compaction might be perturbed in *ERCC6L2*^−/−^ cells (Figures S8, S9, S11 and S12). Such chromatin alterations may play a critical role in regulating accessibility of DNA breaks for nucleolytic processing.^48^ Our data suggest that ERCC6L2 acts independently of the established anti-resection 53BP1-RIF1-REV7-Shieldin axis. With respect to this, we could not detect significant accumulation of ERCC6L2 protein at damage-induced foci or stalled replication forks, which is in contrast to 53BP1 and RIF1.^79^ ERCC6L2 might therefore not engage in “shielding” of DNA ends through a self-imposed physical barrier, but rather use its enzymatic activity to ensure maintenance of the repressive chromatin structure. Mechanistically, this might involve disassembly of secondary structures to facilitate loading of chromatin factors that drive chromatin compaction.

This model is in agreement with the recent studies implicating ERCC6L2 in class switch recombination (CSR).^32^^,^^33^^,^^34^ Importantly, CSR occurs between highly repetitive sequences in switch regions of immunoglobulin heavy chain loci. ERCC6L2’s involvement in CSR might therefore be linked to counteracting secondary structures at switch region repeats and enabling their productive engagement during CSR. This could entail assisting accessibility of DNA for transcription or AID-mediated cytosine deamination, or potentially by promoting juxtaposition of DNA ends for ligation (Figure 7B).

Finally, our mechanistic insights provide a new context for pathological implications associated with ERCC6L2 deficiency. Although IBMFSs are biologically heterogeneous, telomeres feature as one of the main themes in IBMFS pathology, and interesting parallels can be observed between centromeric ERCC6L2 and telomeric RTEL1. This is evidenced by the associated IBMFSs, and by their respective roles in counteracting secondary structures to facilitate replication.^80^^,^^81^ Whether replication stress and/or centromere dysfunction underlie the pathology of ERCC6L2-deficient IBMFS is presently unknown; however, that is an intriguing possibility.

### Limitations of the study

Due to the technical difficulties of establishing the rescue cell lines that stably express wild ERCC6L2 protein and its specific mutant forms, this study did not determine functional consequences of the aPIP-box mutation beyond protein localization. Further studies are therefore needed to address the importance of PCNA interaction in ERCC6L2-mediated activities at DSBs and in DNA replication. Moreover, although our results demonstrate enhanced replication of centromeric DNA in ERCC6L2^−/−^ cells, it would be important to establish whether this is directly caused by the loss of centromeric factors observed in this genomic background. Finally, epigenetic changes observed in ERCC6L2-deficient cells do not suggest a direct role of ERCC6L2 in the deposition of specific histone marks. However, they raise questions about the links between the ERCC6L2 activities and the maintenance of the general chromatin structure, which will need to be examined in greater detail.

## STAR★Methods

### Key resources table

**Table:** 

REAGENT or RESOURCE	SOURCE	IDENTIFIER
**Antibodies**		

β-tubulin	Abcam	ab6046; RRID:AB_2210370
CENP-A	Abcam	ab13939; RRID:AB_300766
CENP-B	Abcam	ab25734; RRID:AB_726801
CENP-C	MBL International	PD030; RRID:AB_10693556
Cyclin A	Santa Cruz	sc-271682; RRID:AB_10709300
γH2AX (S139)	Millipore	05-636; RRID:AB_309864
PCNA	Abcam	ab18197; RRID:AB_444313
RPA32 9H8	Abcam	ab2175; RRID:AB_302873
RPA32/RPA2 (phospho T21)	Abcam	ab61065; RRID:AB_946322
RPA32 (phospho S4/S8)	Bethyl	A300-245A; RRID:AB_210547
RAD51	Abcam	ab88572; RRID:AB_2042762
53BP1	Millipore	MAB3802; RRID:AB_11212586
BrdU	GE Healthcare	RPN202
PICH (ERCC6L)	R&D Systems	H00054821-D01P
TRF1	Abcam	ab10579; RRID:AB_2201461
Histone H1o/H5 3H9	Merck	05-629-I
Tri-Methyl-Histone H3 (Lys27)	Cell Signaling	9733S
Di-Methyl-Histone H3 (Lys27)	Cell Signaling	9728S
Mono-Methyl-Histone H3 (Lys27)	Cell Signaling	84932S
Anti-Histone H3 (acetyl K27)	Abcam	ab4729; RRID:AB_2118291
Histone H3 (tri methyl K9)	Abcam	ab8898; RRID:AB_306848
Anti-dimethyl-Histone H3 (Lys9)	Merck	07–441
Anti-Histone H3 (tri methyl K4)	Abcam	ab8580; RRID:AB_306649
Anti-Histone H4 (di methyl K20)	Abcam	ab9052; RRID:AB_1951942
Anti-HP1 alpha	Abcam	ab109028; RRID:AB_10858495
Polycomb Group 2 (PRC2) Antibody Sampler Kit	Cell Signaling	62083T
Phospho KAP-1 (S824)	Bethyl	A300-767A; RRID:AB_669740
anti-KAP-1	Bethyl	A300-274A; RRID:AB_185559
Strep tag	IBA Life Sciences	2-1507-001
V5 tag	Abcam	ab9116; RRID:AB_307024
Anti-Mouse-HRP	Dako	P0447
Anti-Rabbit-HRP	Dako	P0399
Alexa Fluor 405 Anti-Mouse	Life Technologies	A31553
Alexa Fluor 488 Anti-Mouse	Life Technologies	A11029
Alexa Fluor 594 Anti-Mouse	Life Technologies	A11032
Alexa Fluor 594 Anti-Rabbit	Life Technologies	A11037
Alexa Fluor 647 Anti-Mouse	Life Technologies	A32728
Alexa Fluor 647 Anti-Rabbit	Life Technologies	A21245

**Chemicals, peptides, and recombinant proteins**		

Recombinant human PCNA	Sebesta et al.^82^	N/A
His-tagged human PCNA	Sebesta et al.^82^	N/A
His-tagged ERCC6L2^1053−1247^ fragment	This study	N/A
GST-tagged ERCC6L2^753−819^ fragment	This study	N/A
Biotinylated aPIP ERCC6L2 peptide	Genscript	N/A
Biotinylated aPIP^∗^ ERCC6L2peptide	Genscript	N/A
Biotinylated PIP ZRANB3 peptide	Sebesta et al.^82^	N/A
Biotinylated aPIP^∗^ ZRANB3 peptide	Sebesta et al.^82^	N/A
ERCC6L2^795−816^ aPIP peptide	Genscript	N/A
Phleomycin	Merck	P9564
Hydroxyurea	Merck	H8627
Puromycin	InvivoGen	ant-pr-1
cOmplete, EDTA-free Protease Inhibitor Cocktail	Merck	11873580001
PhosSTOP	Merck	4906845001
Benzonase	Merck	1016970001
4x NuPAGE LDS sample buffer	Life Technologies	NP0007
NuPAGE Novex 4–12% Bis-Tris gel	Life Technologies	WG1402A
DAPI	Merck	D9542
BrdU	Abcam	ab142567
RNASE, DNASE-FREE	Merck	11119915001
Proteinase K, recombinant, PCR grade-	Life Technologies	EO0491
Dynabeads Protein G	Life Technologies	10003D
SPRI SELECT reagent	15676104	Fisher Scientific
16% Formaldehyde (w/v), Methanol-free-10 x	Pierce	28906
Lipofectamine RNAiMAX Reagent	Life Technologies	13778150
Lipofectamine 2000 Transfection Reagent	Life Technologies	11668019
CENPB-Alexa488 Centromere probe	PNA Bio	F3004
Reverse complement of CENPB probe CENPBR-Cy3	PNA Bio	F3009

**Critical commercial assays**		

Click Plus EdU 594 Imaging Kit	Life Technologies	C10639
NEBNext Ultra II End RepairdA-Tailing Module	New England Biolabs	E7546S
NEBNext Ultra II Ligation Module	New England Biolabs	E7595S
Phusion(R) High-Fidelity DNA Polymerase	New England Biolabs	M0530S
QuikChange Lightning Site-Directed Mutagenesis Kit	Agilent Technologies	210518
DNeasy Blood & Tissue Kit	QIAGEN	69504
QIAprep Spin Miniprep Kit	QIAGEN	27104
HiSpeed Plasmid Maxi Kit	QIAGEN	12662

**Deposited data**		

Nascent DNA sequencing data	This study	GEO: GSE226155
ChIP sequencing data	This study	GEO: GSE226155
Crystal structure of human PCNA in complex with ERCC6L2 PIP box peptide	This study	PDB ID: 8COB
Original blots	This study	Mendeley Data https://doi.org/10.17632/pcyj38hp2z.1

**Experimental models: Cell lines**		

U2OS cells	ATCC	HTB-96
hTERT RPE-1 cells	ATCC	CRL-4000
U2OS ERCC6L1^−/−^ cells	This study	N/A
RPE-1 ERCC6L1^−/−^ cells	This study	N/A

**Oligonucleotides**		

sgRNA ERCC6L2 TATGGACACTACATCCATGGAGG	Life technologies	N/A
sgRNA ERCC6L2 GCATAAAAAGGGAACTCGTGAGG	Life technologies	N/A
5′^32^P-labelled oligonucleotide (EMSA ssDNA) CTTCGTTGGAAACGGGATTTCTTCATTTCATGCTA	This study	N/A
EMSA complementary DNA TAGCATGAAATGAAGAAATCCCGTTTCCAACGAAG	This study	N/A

**Recombinant DNA**		

pDONR221 (Gateway vector)	Life technologies	12536017
pDEST-YFP (Gateway vector)	Life technologies	V35820
pEXPR-IBA105	IBA Life Sciences	2-3505-000
pGEX-4T	Merck	GE28-9545-49
pET28a	Merck (Novagen)	69864
pDONR223 STN1	Dharmacon	OHS5894-202505727
pDONR223 PtIP	Dharmacon	OHS1770-202324354
pDONR223 FAM35A	Dharmacon	OHS1770-202310331
YFP ERCC6L2	This study	N/A
YFP ERCC6L2 K165R	This study	N/A
YFP ERCC6L2 PIP^∗^ (Q798A, C804A, F806A)	This study	N/A
YFP ERCC6L2^1−712^	This study	N/A
YFP ERCC6L2^701−1561^	This study	N/A
YFP ERCC6L2^701−1247^	This study	N/A
YFP ERCC6L2^701−1098^	This study	N/A
YFP ERCC6L2^728−1098^	This study	N/A
YFP ERCC6L2^797−1098^	This study	N/A
YFP ERCC6L2^820−1098^	This study	N/A
YFP ERCC6L2^880−1098^	This study	N/A
YFP ERCC6L2^1053−1247^	This study	N/A
YFP ERCC6L2^1248−1561^	This study	N/A
His-tagged ERCC6L2^1053−1247^	This study	N/A
His-tagged PCNA	Sebesta et al.^82^	N/A
GST-tagged ERCC6L2^753−819^	This study	N/A
pEXPR-IBA105 ERCC6L2^701−1247^	This study	N/A
pEXPR-IBA105 ERCC6L2^701−1053^	This study	N/A
YFP ERCC6L2^701−1247^ Q798A	This study	N/A
YFP ERCC6L2^701−1247^ L799A	This study	N/A
YFP ERCC6L2^701−1247^ L801A	This study	N/A
YFP ERCC6L2^701−1247^ Q803A	This study	N/A
YFP ERCC6L2^701−1247^ C804A	This study	N/A
YFP ERCC6L2^701−1247^ F806A	This study	N/A
YFP ERCC6L2 Thr413Cysfs^∗^2	This study	N/A
YFP ERCC6L2 Ile486fs^∗^34	This study	N/A
YFP ERCC6L2 Gln502^∗^	This study	N/A
YFP ERCC6L2 Arg563^∗^	This study	N/A
YFP ERCC6L2 Arg655^∗^	This study	N/A
YFP ERCC6L2 Glu729fs^∗^49	This study	N/A
YFP ERCC6L2 Glu923Argfs^∗^8	This study	N/A
YFP ERCC6L2 Lys985Hisfs^∗^3	This study	N/A
YFP ERCC6L2 Asn1111Lysfs^∗^12	This study	N/A
YFP ERCC6L2 Met1148 Glufs^∗^7	This study	N/A
YFP ERCC6L2 Arg1266^∗^	This study	N/A

**Software and algorithms**		

FastQC	Andrews et al.^83^	N/A
Trimgalore	Krueger et al.^84^	N/A
BOWTIE2	Langmead et al.^85^	N/A
MACS2	Zhang et al.^86^	N/A
MSPC	Jalili et al.^87^	N/A
BEDtools	Quinlan et al.^88^	N/A
MicroCal PEAQ-ITC	https://www.malvernpanalytical.com/	N/A
XDS	https://xds.mr.mpg.de/	N/A
POINTLESS	https://www.ccp4.ac.uk/html/pointless.html#references	N/A
AIMLESS	https://www.ccp4.ac.uk/html/aimless.html	N/A
PHASER	McCoy et al.^89^	N/A
COOT	Emsley et al.^90^	N/A
REFMAC5	Winter et al.; Murshudov et al.^91^^,^^92^	N/A
CellProfiler	Stirling et al.^93^	N/A

### Resource availability

#### Lead contact

Further information and requests for resources and reagents should be directed to and will be fulfilled by the Lead Contact, Dragana Ahel (dragana.ahel@path.ox.ac.uk).

#### Materials availability

Unique reagents generated in this study will be made available on request from the Lead Contact.

### Experimental model and subject details

#### Generation of knockout cell lines

ERCC6L2 knockout (KO) cell lines were generated using CRISPR/Cas9 technology.^94^^,^^95^ Two single-guide RNAs (sgRNAs) sequences, TATGGACACTACATCCATGGAGG and GCATAAAAAGGGAACTCGTGAGG (designed and tested by Dr. Andrew Bassett, formerly of Genome Engineering Oxford), were cloned into the Cas9-expressing vector px459 (a gift from Feng Zhang, Addgene plasmid # 48139) for *ERCC6L2* gene targeting. px459 constructs were transfected into human U2OS or RPE-1 cells (ATCC), and selected with puromycin (U2OS), or blasticidin (RPE-1) for 24h. Clonal cell lines were established from single cells and validated by Sanger sequencing. Selected cell lines were found to contain the following mutations:

WTGCATAAAAAGGGAACT-CGTGAGG
U2OS ERCC6L2^−/−^1 GCATAAAAAGGGAACTCCGTGAGG.
WT GCATAAAAAGGGAACTCGTGAGG.
U2OS ERCC6L2^−/−^2 GCATAAAAAGGGAACTC-TGAGG.
U2OS ERCC6L2^−/−^2 GCATAAAAAGGGAACT--TGAGG.
WT TATGGACACTACATCCA--TGGAGG.
U2OS ERCC6L2^−/−^3 TATGGACACTACATCCAA-TGGAGG.
U2OS ERCC6L2^−/−^3 TATGGACACTACATCCAACTGGAGG.
WT TATGGACACTACATCCA-TGGAGG.
U2OS ERCC6L2^−/−^4 TATGGACACTACATCCAATGGAGG.
WT GCATAAAAAGGGAACTCGTGAGG.
RPE-1 ERCC6L2^−/−^1 GCATAAAAAGGGAACT-GTGAGG.
WT TATGGACACTACATCCATGGAGG.
RPE-1 ERCC6L2^−/−^2 TATGGACACTACATCC-TGGAGG.
RPE-1 ERCC6L2^−/−^2 TATGGACACTACATC-ATGGAGG.

#### Maintenance

The cell cultures were maintained in DMEM (Sigma) supplemented with 10% FBS (GIBCO) and penicillin-streptomycin (100 U/mL, GIBCO) at 37°C with 5% CO2.

### Method details

#### Plasmids

Full-length human *ERCC6L2* was generated using gene synthesis (GeneArt, LIfe Technologies) and cloned into pDONR221 entry vector (Thermo Fisher Scientific). Point mutations were introduced using the QuickChange II site-directed mutagenesis kit (Agilent). For the expression of YFP-tagged proteins, ERCC6L2 constructs were cloned by Gateway LR reaction to pDEST-YFP/FRT/TO. For the expression of the Strep-tagged ERCC6L2 proteins in human cells, the ERCC6L2 constructs were cloned into pEXPR-IBA105 (IBA Life Sciences). pGEX-4T was used for the expression of the GST-tagged ERCC6L2 constructs in E. coli. Human PCNA was cloned into NcoI and XhoI sites of pET28a vector for the expression of the untagged protein, and into NdeI and XhoI sites for the expression of the protein with N-terminal His-tag. Constructs for STN1, PtIP, and FAM35A were obtained from Dharmacon.

#### Cell extracts and immunoprecipitation

Cell lysates were prepared by resuspending cells in lysis buffer (50 mM Tris pH 8, 150mM NaCl, 1 mM DTT (Fisher Scientific), 1% Triton X-, supplemented with one protease inhibitor tablet per 30 mL total volume (Roche) and 500 U/μl Benzonase (Sigma-Aldrich)). Samples were then sonicated thoroughly (4 rounds for 30 s each on full power using a bench-top sonicator (MSE Soniprep 150)) and placed on a rotor wheel at 4°C for 45 min. Lysates were centrifuged in a bench-top microcentrifuge (Eppendorf) at 15,000 x g for 20 min at 4°C. For immunoprecipitation, cell extracts derived from control cell lines and cell lines expressing Strep-tagged ERCC6L2 constructs were applied to StrepTactin Sepharose beads (IBA Life Sciences). The beads were then extensively washed with lysis buffer. Immunocomplexes were subsequently eluted with 25 nM biotin in Tris pH 8, boiled in SDS-PAGE loading buffer, and analyzed by Western blotting.

For preparation of whole cell extracts cells were resuspended in lysis buffer (50 mM Tris pH 8, 150mM NaCl, 1 mM DTT, 1% Triton X-, 1% SDS, protease inhibitors, phosphatase inhibitors (Roche, 1 tablet per 10 mL), and boiled for 5 min. After cooling to room temperature benzonase was added and extracts were incubated at for 30 min. They were then boiled, centrifuged at maximum speed for 5 min, and prepared for SDS-PAGE. For preparation of chromatin extracts, cells were pre-extracted with PBS containing 0.5% Triton X-, washed in PBS containing 0.25% Triton X-, and then processed using protocol for preparation of whole cell extract.

#### Clonogenic survival assay

Cells were seeded in 6-well plates (approximately 700 cells/well) and either left untreated or treated with the indicated concentrations of genotoxic agents. Cells were then grown for 11 days to allow colony formation. Colonies were stained with 5 mg/ml crystal violet (Sigma-Aldrich) solution in 25% methanol for 30 min. Pictures were taken using a Nikon D3200 camera and images were analyzed using FIJI software. FIJI’s ‘Analyze Particles’ function was used to quantify numbers of colonies. Survival was calculated relative to untreated cells. Each experiment was performed in technical triplicates, and data shown represent combined data from at least three independent experiments.

#### Cell cycle analysis by flow cytometry

U2OS cells were grown to around 70% confluence in 10 cm dishes and treated with the indicated doses of etoposide for 48 h. Cells were then pulse-labelled for 3h with EdU. Samples were then processed for flow cytometry by using the Click-iT EdU Flow Cytometry Assay Kit (Life Technologies). Incorporated EdU was detected by using click chemistry with Alexa Fluor 647 dye, while DNA was stained with FxCycle Violet (Life Technologies), as per the manufacturer’s instructions. Flow cytometry was performed on a Cytek DxP. Gating was applied to exclude dead cells and doublets from the analysis, and 50,000 cells within the gating parameters were analyzed per condition. Data were analyzed using FlowJo software (FlowJo).

#### siRNA transfection

Previously characterised siRNAs were used in this study:

siCTRL CGUACGCGGAAUACUUCGA(dTdT)^59^
siBLM CCGAAUCUCAAUGUACAUAGA(dTdT)^72^
siBRCA1 GGAACCUGUCUCCACAAAG(dTdT)^59^
siCtIP GCUAAAACAGGAACGAAUC(dTdT)^59^
siRIF1 AGACUUGUCUCAGAUAUAA(dTdT)^59^
siREV7 ON-TARGETplus L-003272-00-0005 (Dharmacon)
siFAM35A UCAACAUUAUGCGCUUGUA(dTdT)^59^
siC20orf196 GCGUGUGACAUAAGAGAUU(dTdT)^59^
si53BP1 GAAGGACGGAGUACUAAUA(dTdT)^59^
siPtIP ACGUGAUCGGAGUGUGUAUAA^59^
siSTN1 GCUUAACCUCACAACUUAA(dTdT)^96^
siCTC1 ON-TARGET plus L-014585-01-0005 (Dharmacon)
siBRCA2 GAAGAAUGCAGGUUUAAUATT^97^
siRAD51 GACUGCCAGGAUAAAGCUU(dTdT)^97^
siCENPA ON-TARGETplus L-003249-00-0005 (Dharmacon)
siCENPB ON-TARGETplus L-003250-00-0005 (Dharmacon)
siHJURP ON-TARGETplus L-015443-00-0005 (Dharmacon)

Individual siRNAs were purchased from Dharmacon or from Sigma Aldrich. Cells were seeded into 24-well plates and transfected with 24 or 36 nM siRNA after 24 and 48 h using Lipofectamine RNAiMAX (Life Technologies).

#### Immunofluorescence

U2OS cells (ATCC) were grown in 24 well plates. Where indicated, cells were transfected with the YFP constructs using or with siRNAs. Cells were fixed in PTEMF buffer (20 mM PIPES pH 6.8, 0.2% Triton X-100, 1 mM MgCl2, 10 mM EGTA and 4% paraformaldehyde) for 15 min, permeabilized with 0.5% Triton X- for 5 min, washed in PBS, and further incubated in 2% BSA for 30 min. Primary and secondary antibodies were diluted in 2% BSA. Cells were stained with primary antibodies for 1 h, washed with PBS, and incubated with Alexa Fluor conjugated secondary antibodies for another 1 h. They were then washed again in PBS, and stained with DAPI. Samples were analyzed by confocal microscopy using Olympus FV1200 or Zeiss 880 Airyscan microscopes, or using EVOS M7000 Imaging System.

For quantitative analysis of pRPA, RAD51, γH2AX, and EdU foci, non-overlapping images were acquired using a 20x objective lens. Image analysis was performed using CellProfiler.^98^ For quantification of TRF1-and CENP-C-positive micronuclei, random non-overlapping images were acquired, and Z-stacks were further collected for each micronucleus to examine the presence of TRF1 and CENP-C foci.

Drug treatments were as follows: for analysis of UFBs, cells were treated with 25 μg/mL phleomycin and allowed to recover for 2 days before fixation. 10 μM VE-821 was added to the media where indicated. For quantitative analysis of damage-induced foci, 24 h after the second siRNA transfection cells were exposed to 25 μg/mL phleomycin for 1 or 2 h, after which they were allowed to recover for 6 h before fixation. Where indicated, cells were incubated with 50 μM B02, 10 μM ETP-46464, VE-821, KU-60019 or KU-55933 (all Sigma) after exposure to phleomycin. For visualisation of nascent DNA synthesis, cells were pulsed with EdU as indicated and stained using a Click-iT EdU Alexa Fluor 594 imaging kit (Life Technologies) according to the manufacturer’s protocol.

For native BrdU staining, cells were incubated with 30 mM BrdU for 24 h, and treated as indicated. Fixation was performed as described above, and resected ssDNA was detected under native conditions by incubation with mouse anti-BrdU antibody.

For quantitative image-based cytometry single-cell analysis (QIBC), cells were pulsed with EdU for 15 min where indicated and stained using a Click-iT EdU Alexa Fluor 594 imaging kit, and CENP-A, CENP-B or CENP-C antibodies. DNA was visualised using DAPI. Non-overlapping images were acquired using a 20x objective lens and analyzed using CellProfiler. Cell cycle distribution profiles were obtained by plotting nuclear DAPI intensity vs. nuclear EdU intensity. Alternatively, cell cycle distribution profiles were obtained by using Cyclin A staining instead of EdU.

#### Live-cell imaging by laser microirradiation

U2OS cells were grown in glass-bottomed 24 well plates and transfected with YFP-ERCC6L2 constructs using Lipofectamine 2000 (Life Technologies). Transfected cells were then incubated with 10 μM BrdU for 16 h at 37°C. Laser microirradiation was carried out on an Olympus FV1200 confocal microscope equipped with an environmental chamber. Localised DNA damage was induced in cells showing YFP-tagged protein expression using a 405 nm laser. Recruitment of the proteins was monitored by live cell imaging at 488 nm.

#### Protein purification

Untagged PCNA expressed from pET28a vector was used for crystallisation of PCNA:aPIP(ERCC6L2) complex. Expression was induced with 0.4 mM IPTG at 30°C for 4 h. Cells were lysed in lysis buffer (50 mM Tris-HCl pH 8, 150 mM NaCl, 1 mM DTT, 2 mg/mL lysozyme, protease inhibitor cocktail (Roche) and 25 U/ml of benzonase (Sigma-Aldrich)) by applying three passages through a French-press at 15,000 Psi. The extract was centrifuged for 90 min at 35,000 g, and the supernatant was then applied to a 10 mL Q-sepharose column equilibrated to Buffer A (50 mM Tris-HCl pH 8, 150 mM NaCl and 1 mM DTT). Elution was performed with 50 mM Tris-HCl pH 7.5, 1 mM DTT and a gradient of 150–550 mM NaCl. Fractions containing PCNA were then purified over a 1 mL S-sepharose column equilibrated to Buffer A, and the flow-through (containing PCNA) was applied onto a 5 mL Hi-Trap Heparin column equilibrated with Buffer A. PCNA was again collected in the flow-through, which was then loaded onto a 5 mL Q-sepharose column equilibrated with Buffer A. PCNA was eluted with a gradient of 50–500 mM NaCl, and fractions containing PCNA were pooled, concentrated and loaded onto a Superdex S-200 (16/600) column equilibrated with storage buffer (20 mM Tris-HCl pH 8, 100 mM NaCl, 1 mM DTT). Purified PCNA was concentrated to ∼20 mg/mL before being used to set-up crystallisation trials.

His-tagged PCNA was expressed from the pET28a vector in Rosetta *E. coli* using the same conditions as the untagged PCNA. Cells were lysed in lysis buffer (50 mM Na-Phosphate pH 8, 500 mM NaCl, 1 mM β-mercaptoethanol, 10 mM imidazole, 2 mg/mL lysozyme, protease inhibitor cocktail (Roche) and 25 U/ml of benzonase (Sigma-Aldrich)). The extract was applied onto Ni-NTA beads (Qiagen) equilibrated with lysis buffer, and eluted by increasing the concentration of imidazole to 500 mM. The protein was subsequently applied to a 5 mL Q-sepharose column equilibrated to the PBS buffer containing 1 mM β-mercaptoethanol, and eluted in the same buffer with 150–500 mM NaCl gradient. PCNA containing fractions were pooled and purified over a Superdex S-200 (16/600) column equilibrated to the PBS buffer containing 1 mM β-mercaptoethanol. For ITC measurements, PCNA was concentrated to ∼600 μM and extensively dialyzed against the PBS buffer containing 1 mM β-mercaptoethanol, along with the ligand peptides.

His-tagged ERCC6L2^1053−1247^ fragment was expressed from the pET28a vector in *E. coli* using the same conditions as the His-tagged PCNA. Cells were lysed in lysis buffer (50 mM Tris-HCl pH 8, 500 mM NaCl, 1 mM DTT, 10 mM imidazole, 2 mg/mL lysozyme, protease inhibitor cocktail (Roche) and 25 U/ml of benzonase (Sigma-Aldrich)) and sonicated as described above. The extract was cleared by centrifugation at 35,000 g for 30 min, applied onto Ni-NTA beads (Qiagen), and extensively washed with the wash buffer (50 mM Tris-HCl pH 8, 500 mM NaCl, 1 mM DTT, 30 mM imidazole). Protein was eluted with elution buffer (50 mM Tris-HCl pH 8, 500 mM NaCl, 1 mM DTT, 500 mM imidazole), and applied onto a Superdex S-200 (16/600) column. Fractions containing pure His-tagged ERCC6L2^1053−1247^ fragment were pooled, concentrated and stored at −80°C.

GST-tagged ERCC6L2^753−819^ fragment was expressed from pGEX4T vector in E. coli. Expression was induced with 0.4 mM IPTG at 30°C for 4h. Cells were lysed in PBS buffer supplemented with 1 mM DTT, protease inhibitor cocktail (Roche) and 25 U/ml of benzonase (Sigma-Aldrich) and sonicated as described above. The extract was cleared by centrifugation at 35,000 g for 30 min, and applied onto GST-beads (GE healthcare) equilibrated in PBS containing 1 mM DTT. After extensive washing with 50 mM Tris-HCl pH 8, 150 mM NaCl, 1 mM DTT, beads were directly used in the PCNA pull down experiment.

#### PCNA pull-downs

Custom synthetic biotinylated peptides were synthetised by Genscript and dissolved in 50 mM Tris-HCl pH 8, 150 mM NaCl. The peptides were immobilised on magnetic streptavidin beads (Life Technologies), which were then washed with 50 mM Tris-HCl pH 8, 150 mM NaCl, 1 mM DTT and 0.2% Triton X-. The beads were then incubated with purified His-tagged PCNA for 1 h at 4°C, and subsequently washed with the same buffer. Finally, the beads were boiled in the SDS-PAGE loading buffer, and analyzed by anti-PCNA Western blotting (1:1000 dilution).

Additionally, GST-tagged ERCC6L2^753−819^ fragment was immobilised on GST-beads and incubated with purified His-tagged PCNA for 1 h at 4°C in 50 mM Tris-HCl pH 8, 150 mM NaCl, 1 mM DTT and 0.2% Triton X-. The beads were then extensively washed with the same buffer, and prepared for Western-blotting.

#### Electrophoretic mobility shift assay (EMSA)

EMSA was performed with either single-stranded DNA or double-stranded DNA substrates, derived from α-satellite DNA sequence. A 5′ ^32^P-labelled oligonucleotide (5′-CTTCGTTGGAAACGGGATTTCTTCATTTCATGCTA -3′) was used for analysis of ssDNA binding, whereas dsDNA was prepared by mixing a 1.2-fold excess of unlabelled oligonucleotide (5′-TAGCATGAAATGAAGAAATCCCGTTTCCAACGAAG-3′) over the 5′ 32P-labelled oligonucleotide. The oligonucleotides were heated to 94°C for 3 min, and annealed by slow cooling of reactions from 94°C. Purified His-tagged ERCC6L2^1053−1247^ fragment (11 μM–176 μM) was incubated with radioactively labeled substrate (2.5 nM) at room temperature in 15 μL of reaction buffer (50 mM Tris-HCl pH 7.5, 5% glycerol, 4 mM EDTA, 1 mM DTT and 0.1 mg/mL BSA) for 30 min. After the incubation, the reaction mixtures were resolved in a 5% native polyacrylamide gel in 0.5 × TBE buffer (45 mM Tris-borate, 1 mM EDTA; pH 7.5) at 4°C. Resolved gels were dried and visualised by autoradiography.

#### Isothermal titration calorimetry

PCNA and the ERCC6L2 aPIP peptide (ERCC6L2 amino acids 795–816) were extensively dialyzed against PBS containing 1 mM β-mercaptoethanol. Molar absorptivity calculated from the amino acid sequence was used to determine the concentration of the peptide by measuring absorption at 205 nm.

The measurements were performed at 25°C using MicroCal PEAQ-ITC (Malvern). 15 μM solution of PCNA was added to the reaction chamber, while the 215 μM solution of ERCC6L2 aPIP peptide was injected in 2 μL steps (20 steps in total). Results were analyzed using MicroCal PEAQ-ITC Analysis Software. The free Gibbs energy (ΔG), Enthalpy change (ΔH) and stoichiometry (N) were determined by Levenberg–Marquardt curve-fitting method employing single set of independent binding sites model. Association constant (KA) was determined using the equation ΔG = −RTln (KA). The association constant (KA) is the inverse function of the dissociation constant KD. Finally, the entropy difference (−TΔS) was obtained from the following equation: ΔG = ΔH–TΔS.

#### Crystallisation, data collection and structure solution

Peptide derived from ERCC6L2 amino acid sequence 795–816 was used for crystallisation of the PCNA:aPIP(ERCC6L2) complex. 1:10 M ratio of PCNA:aPIP(ERCC6L2) peptide was used to obtain co-crystals. Co-crystals grew in 0.1M Bis-Tris pH 5.5; 25% (w/v) PEG 3350. They were cryoprotected by a 2–5 sec soak in crystallisation solution with 20% glycerol, before being vitrified by submersion in liquid nitrogen. X-ray data were collected at beamline ID30A-1 of the European Synchrotron Radiation Facility (71 Avenue des Martyrs, 38000 Grenoble, France).^99^ Data collection statistics are shown in Table S1. The PCNA:aPIP(ERCC6L2) structure was solved by molecular replacement with a native human PCNA structure (PDB code: 8COB) as the molecular replacement model.

X-ray data were processed using XDS, POINTLESS and AIMLESS. PHASER was used for phasing by molecular replacement. Model building was carried out with COOT and real space refinement with REFMAC5,^91^^,^^92^ coupled with automatically generated local non-crystallographic symmetry restraints and TLS refinement.

#### Replication combing assay

Control and *ERCC6L2*^−/−^ cells were labeled with 100 μM CldU, followed by 100 μM IdU in the presence of 10 μM Polα inhibitor CD437. Each pulse lasted 40 min. Cells were then trypsinised and embedded in agarose plugs using a kit from Genomic Vision according to the manufacturer’s instructions. DNA was extracted and purified from the agarose plugs, and stretched onto silanised coverslips with the molecular combing system (Genomic Vision). CldU was detected with rat anti-BrdU antibody (Abcam, BU1/75) and Alexa Fluor 594 conjugated secondary antibody (Thermo Fisher Scientific). ldU was detected with mouse anti-BrdU antibody (Becton Dickinson, BD44) and Alexa Fluor 488 conjugated secondary antibody (Thermo Fisher Scientific). Images were processed using FiberStudio software (Genomic Vision). Replication fork speed was estimated from the length of the IdU or CldU tracks labeled during a 40 min period, with 1 μm corresponding to approximately 2 kb DNA. Fork symmetry was estimated as a ratio of individual IdU to CldU track lengths. More than 1500 tracks were analyzed per condition.

For nascent DNA degradation assay, cells were sequentially incubated with 100 μM CldU and 100 μM IdU. This was followed by a 4 h incubation with 3 mM hydroxyurea. Cells were then processed as described above. Fork stability was estimated as a ratio of individual IdU to CldU track lengths. More than 700 tracks were analyzed per condition.

#### Centromere CO-FISH

Centromere CO-FISH was performed essentially as described by,^37^ with small changes. Briefly, WT and *ERCC6L2*^−/−^ cells were cultured in media containing 7.5 μM BrdU and 2.5 μM BrdC for 17 h, and then treated with 0.1 μg/mL colcemid. Cells were trypsinised and incubated in 75 mM KCl at 37°C for 15 min, after which they were centrifuged and the supernatant was removed. Cold fixative (3:1 methanol/acetic acid) was then added dropwise while the cells were gently mixed on a vortex. Metaphase spreads were prepared by dropping the cells onto glass slides, which were then incubated for 3 min on a humidified 50°C heating block. The slides were re-hydrated in PBS for 5 min and treated with RNase A (0.5 μg/mL in PBS) (Sigma) or 15 min at 37°C. They were then stained with 0.5 μg/mL Hoechst 33258 (Sigma) in 2x SSC for 15 min at room temperature, and irradiated with 6 × 10^3^ J/m^2^ using a Stratalinker 1800 UV irradiator. BrdU/BrdC labeled DNA strands were then digested with 10 U/ml Exonuclease III (Promega M1811) at 37°C for 45 min, washed in PBS for 5 min, and dehydrated by consecutive incubation with 75%, 95% and 100% ethanol. The slides were then air-dried and incubated with hybridisation solution (70% formamide, 10 mM TrisHCl pH 7.2, and 0.5% blocking reagent (Roche, diluted from 10% stock prepared in 100 nM maleic acid pH 7.5 and 150 mM NaCl) at room temperature for 30 min. CENP-BR-Cy3 PNA probe (reverse complement of CENP-B probe, PNA Bio F3009) was then boiled at 80°C for 3 min and a 0.5 μM working solution (prepared by diluting the probe in hybridisation solution) was applied to the slides, which were then hybridised at room temperature for several hours or overnight. The slides were washed briefly in wash buffer 1 (70% formamide, 10 mM Tris-HCl, 0.1% BSA), before the 2 h hybridization with CENP-B-Alexa 488 PNA probe (PNA Bio F3004). Slides were then washed twice in wash buffer 1 for 15 min, and then for 5 min in wash buffer 2 (0.1 M Tris-HCl, pH 7.0/0.15 M NaCl/0.08% Tween 20). DNA was then stained in DAPI solution prepared in water (Sigma). After another 5 min in wash buffer 2, the slides were dehydrated by incubation with 75%, 95% and 100% ethanol and air-dried. Finally, they were mounted in antifade reagent (ProLong Gold, Invitrogen) and imaged.

#### Chromatin immunoprecipitation-sequencing (ChIP-seq)

ChIP was performed similarly to a protocol described in.^100^ Briefly, cells were cross-linked at room temperature for 10 min using 1% formaldehyde. Cross-linking reaction was quenched by the addition of 125 mM glycine for 5 min, after which cells were washed in PBS. Cells were then resuspended in the lysis buffer (50 mM Tris pH 7.5, 500 mM NaCl, 3mM CaCl_2_, 1% Triton X-, 0.1% SDS, 0.1% Sodium Deoxycholate, Protease Inhibitors), and chromatin was digested by the micrococcal nuclease for 20–25 min at 37°C. Reactions were stopped by the addition of 10mM EDTA and 20 mM EGTA, and then briefly sonicated. Samples were then cleared by centrifugation at 4°C, and a fraction of the chromatin extract was saved for DNA purification (input). Chromatin extracts were diluted to 50 mM Tris pH 7.5, 250 mM NaCl, 1% Triton X-, 0.1% SDS, 0.1% sodium deoxycholate, protease inhibitors), and incubated with CENP-B antibody for 1 h. Protein G Dynabeads (Life Technologies) were washed and added to the samples, which were then incubated overnight at 4°C. Following immunoprecipitation, beads were pelleted using a magnetic rack, and washed 3 times in 50 mM Tris pH 7.5, 250 mM NaCl, 2 mM EDTA, 1% Triton X-, 0.1% SDS, 5 times in 50 mM Tris pH 7.5, 500 mM NaCl, 2 mM EDTA, 1% Triton X-, 0.1% SDS, once in 10 mM Tris-HCl pH 7.5, 0.25 M LiCl, 1% NP-40, 1% Sodium Deoxycholate, 1 mM EDTA, and once in 10 mM Tris pH 7.5, 1 mM EDTA. Beads were then resuspended in 10 mM Tris pH 7.5, 1 mM EDTA, 1% SDS and incubated at 65°C for 15 min. Eluted DNA and input samples were treated with RNase A at 37°C for 30 min, and with Proteinase K at 65 C overnight in a buffer containing 50 mM Tris HCl pH7.5, 300 mM NaCl and 1% SDS. DNA was then isolated using SPRIselect beads (Beckman Coulter) and used for construction of libraries. Briefly, NEBNext Ultra DNA Library Prep Kit (New England Biolabs) was used for adaptor ligation. DNA was then purified using SPRIselect beads, and amplified for 15 cycles with Illumina indexing primers using NEBNext Ultra II kit and NEBNext Multiplex Oligos for Illumina kit as instructed by the manufacturer’s protocol. Amplified libraries were cleaned using SPRIselect beads and used for sequencing on NovaSeq platform.

#### Genome wide analysis of replication by nascent DNA sequencing

Nascent DNA sequencing of control and ERCC6L2-deficient U2OS cell lines was performed by adapting a repli-seq method, described in detail in.^101^ Briefly, we pulsed the cells with 100 μM BrdU for 2 h and collected them for isolation of genomic DNA with DNeasy Blood & Tissue Kit (Qiagen), according to the manufacturer’s instructions. All samples were prepared in triplicates. Purified DNA was sonicated to obtain fragments of average size of 200-300 bp. DNA was then concentrated using SPRIselect beads (Beckman Coulter) and eluted in TE buffer. 1 μg of DNA was used with NEBNext Ultra DNA Library Prep Kit (New England Biolabs) and NEBNext Multiplex Oligos for Illumina kit for adaptor ligation, as instructed by the manufacturer’s protocol. DNA was then purified using SPRIselect beads, and eluted in water. BrdU labeled *E. coli* genomic DNA was processed in the same way for library preparation, and used as a spike control for BrdU immunoprecipitation. DNA was denatured at 95°C for 5 min and quickly cooled on ice. It was then incubated anti-BrdU antibody (BD347580) for 2 h in IP buffer (PBS, 0.0625% Triton X-100), after which Protein G Dynabeads were added for further 2 h incubation. Beads were pelleted and washed in IP and TE buffers using a magnetic rack, after which immunoprecipitated DNA was eluted by incubating beads in elution buffer (TE, 1% SDS) for 15 min at 65°C. DNA was then purified using SPRIselect beads, and amplified for 15 cycles with Illumina indexing primers using NEBNext Ultra II kit and NEBNext Multiplex Oligos for Illumina kit. Amplified libraries were cleaned using SPRIselect beads and used for sequencing on NovaSeq 6000 platform.

#### Data analysis

For ChIP-seq, the unprocessed reads were first run through FastQC^83^ to check the quality of the sequencing. Then the reads were trimmed using Trimgalore^84^ and mapped to the human genome using BOWTIE2.^85^ The mapped reads are then used to call enrichment peaks using MACS2.^86^ Once called, the peaks are filtered according to the stringent threshold (using the -log10 of the FDR p value of 2.8e^−28^). Peaks common to all 3 replicates in each group are obtained by merging peaks between samples using MSPC.^87^

For nascent DNA sequencing, the analysis was done in several steps: preprocessing and QC, mapping, deduplication, peak calling, replicates merging and determination of group specific peaks. First, the fastq files were checked using FastQC^83^ and trimmed using TrimGalore.^84^ Once trimmed the reads were mapped using BOWTIE2^85^ and the UCSC reference genome for human (HG38 patch 12). The *E.coli* reference was downloaded from the NCBI website (https://www.ncbi.nlm.nih.gov/nuccore/NC_002695.2). Once the alignment was completed, duplicates reads (mostly PCR duplicates) were removed to improve peak calling using Picard tools.^102^ Following deduplication, peak calling was performed using MACS2^86^ for each individual sample. The fragments size determined by MACS2 was around 300 bp (most samples were between 270 and 310). Peaks from replicate samples were then combined using MSPC^87^ to generate consensus peaks for each sample group. In further steps, consensus peaks were compared between different sample groups to exclude common peaks and generate lists of unique peaks. This was performed using BEDtools.^88^ UCSC browser was used to display the position of peaks against the genome as a custom tracks.

### Quantification and statistical analysis

Statistical analyses were performed using excel. The N number for each experiment and details of statistical analyses are described in the Figure legends. Results are presented using bar charts (showing means with standard deviations), or using box and whisker plots to show distribution of a set of data. In box and whisker plots data is divided into quartiles, with a box drawn between the first and third quartiles; an additional line is drawn along the second quartile to mark the mean. Vertical lines extending outside of the box (“whiskers”) indicate variability outside the upper and lower quartiles, and any point outside those lines is considered an outlier. Significant differences are indicated in the figures by ^∗^p ≤ 0.05, ^∗∗^p ≤ 0.01, ^∗∗∗^p ≤ 0.001, ^∗∗∗∗^p ≤ 0.0001, NS not significant. Notable non-significant differences are indicated in the figures by NS.

## Data Availability

• Nascent DNA and ChIP sequencing data generated during this study have been deposited at Gene Expression Omnibus repository, https://www.ncbi.nlm.nih.gov/geo and are publicly available as of the date of publication. Accession numbers are listed in the key resources table. Coordinates and structure factors were deposited in the Protein Data Bank with accession code 8COB. Original data are available at Mendeley Data (https://doi.org/10.17632/pcyj38hp2z.1). • This paper does not report original code. • Any additional information required to reanalyze the data reported in this paper is available from the lead contact upon request.

## Supplemental information

Document S1. Figures S1–S13 and Table S1

Document S2. Article plus supplemental information
