# Supplementary material for: ERCC6L2 mitigates replication stress and promotes centromere stability
Source: Cell Rep. 2023 Apr 3;42(4):112329. doi: 10.1016/j.celrep.2023.112329 (PMC12003248; doi:10.1016/j.celrep.2023.112329)
Supplement: Document S1. Figures S1–S13 and Table S1 [file mmc1.pdf]

Cell Reports, Volume *42*

Supplemental information

ERCC6L2 mitigates replication stress and promotes centromere stability

Christopher J. Carnie, Lucy Armstrong, Marek Sebesta, Antonio Ariza, Xiaomeng Wang, Emily Graham, Kang Zhu, and Dragana Ahel

Table S1. Summary of crystallographic statistics.

	**PCNA:aPIP(ERCC6L2)**
**PDB accession code**	8COB
**Data Collection**	
Synchrotron/beam line	ESRF/ID30A-1
Wavelength (Å)	0.96600
Space group	$P2_1\ 2_1\ 2_1$
a (Å)	67.69
b (Å)	109.08
c (Å)	139.40
α (°)	90.00
β (°)	90.00
γ (°)	90.00
Content of asymmetric unit	3
Resolution (Å) [a]	48.56 – 2.73 (2.86 – 2.73)
R_{sym} (%)[a],[b]	10.7 (47.3)
I/σ(I)	10.5 (3.0)
Completeness (%)[a]	99.8 (97.9)
Redundancy[a]	5.0 (4.8)
$CC_{1/2}$ (%)[a]	99.5 (50.0)
Unique reflections[a]	28128 (3678)
**Refinement**	
R_{cryst} (%)[c]	19.7
R_{free} (%)[d]	25.4
Rmsd bond length (Å)	0.014
Rmsd bond angle (°)	1.61
Number of atoms	6252
Average B factor (Å^2)	63.0
**Ramachandran plot**	
Favoured (%)	97.13
Allowed (%)	2.19
Disallowed (%)	0.68

a Data for the highest resolution shell are given in parentheses.
b R_{sym} = Σ|/-</>|/Σ/, where / is measured density for reflections with indices *hkl*.
c R_{cryst} = Σ||Fobs| - |Fcalc||/Σ|Fobs|.
d R_{free} has the same formula as R_{cryst}, except that calculation was made with the structure factors from the test set.

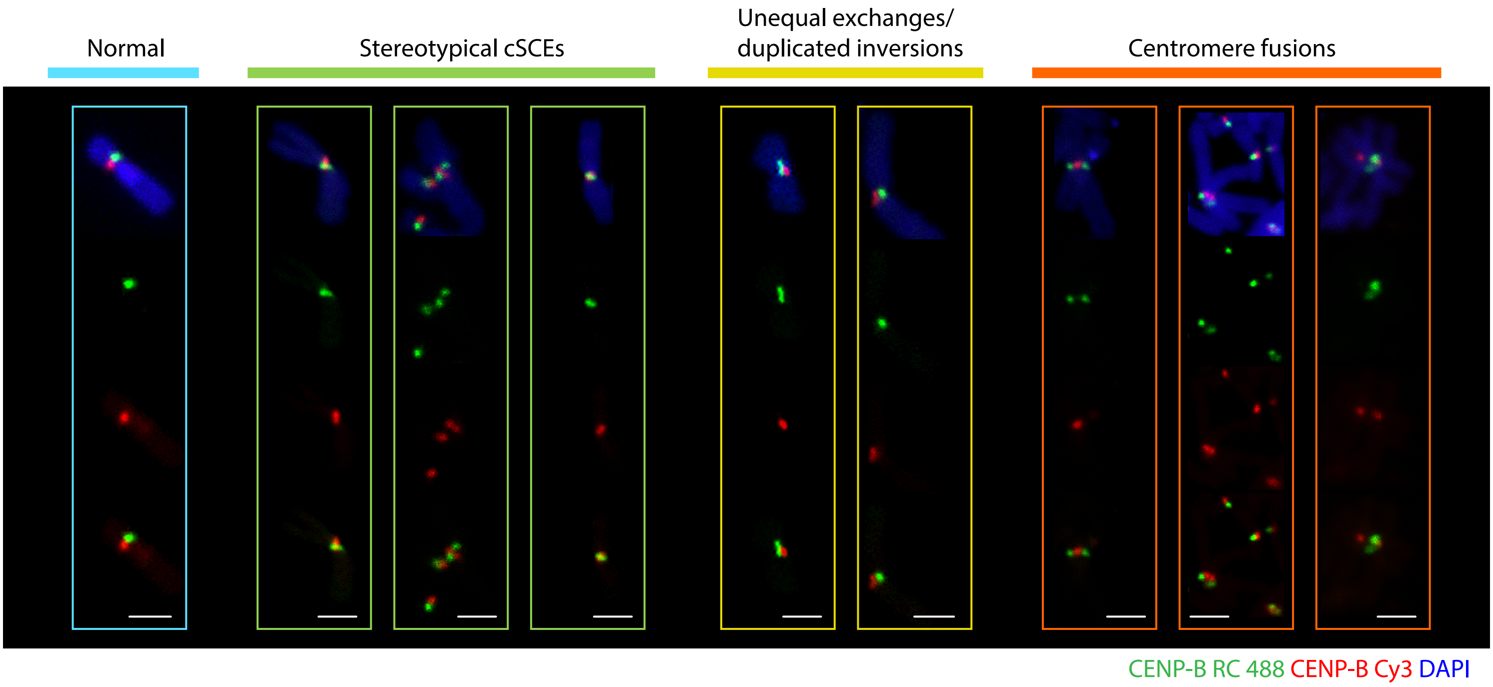

Figure S1. Examples of normal and aberrant centromeric CO-FISH patterns observed in hTERT-RPE1 cell lines. Related to Figure 1 I-K. Scale bar: 2µm.

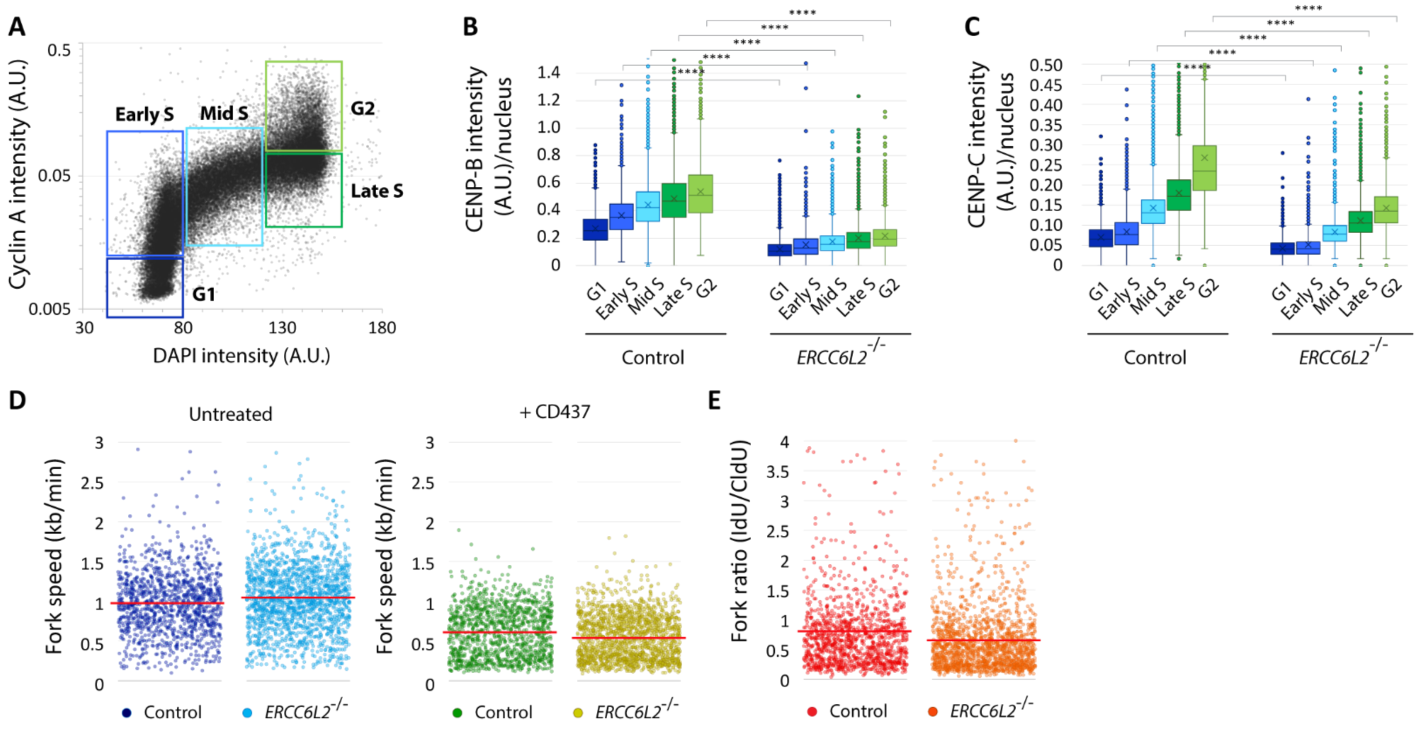

Figure S2. Effects of ERCC6L2 deficiency on centromeric chromatin and DNA replication. Related to Figure 2.
(A) QIBC assay using cyclin A and DAPI intensities. Subpopulations of cells in distinct phases of the cell cycle are shown in coloured frames. N(cells)>50,000. (B) Quantification of CENP-B intensities in different cell cycle subpopulations, identified as in A. (C) Quantification of CENP-C intensities, as in B. (D) Quantification of replication fork speed calculated from the length of CldU labelled tracks (in kb) (left, untreated condition), and IdU labelled tracks (right, in the presence of 10 mM Polα inhibitor CD437) during a 40 min period. N(tracks)>1500. (E) Quantification of replication fork speed symmetry calculated as a ratio of individual IdU to CldU track lengths. N(tracks)>1500.
B and C: ****P ≤ 0.0001.

Figure S3.

A

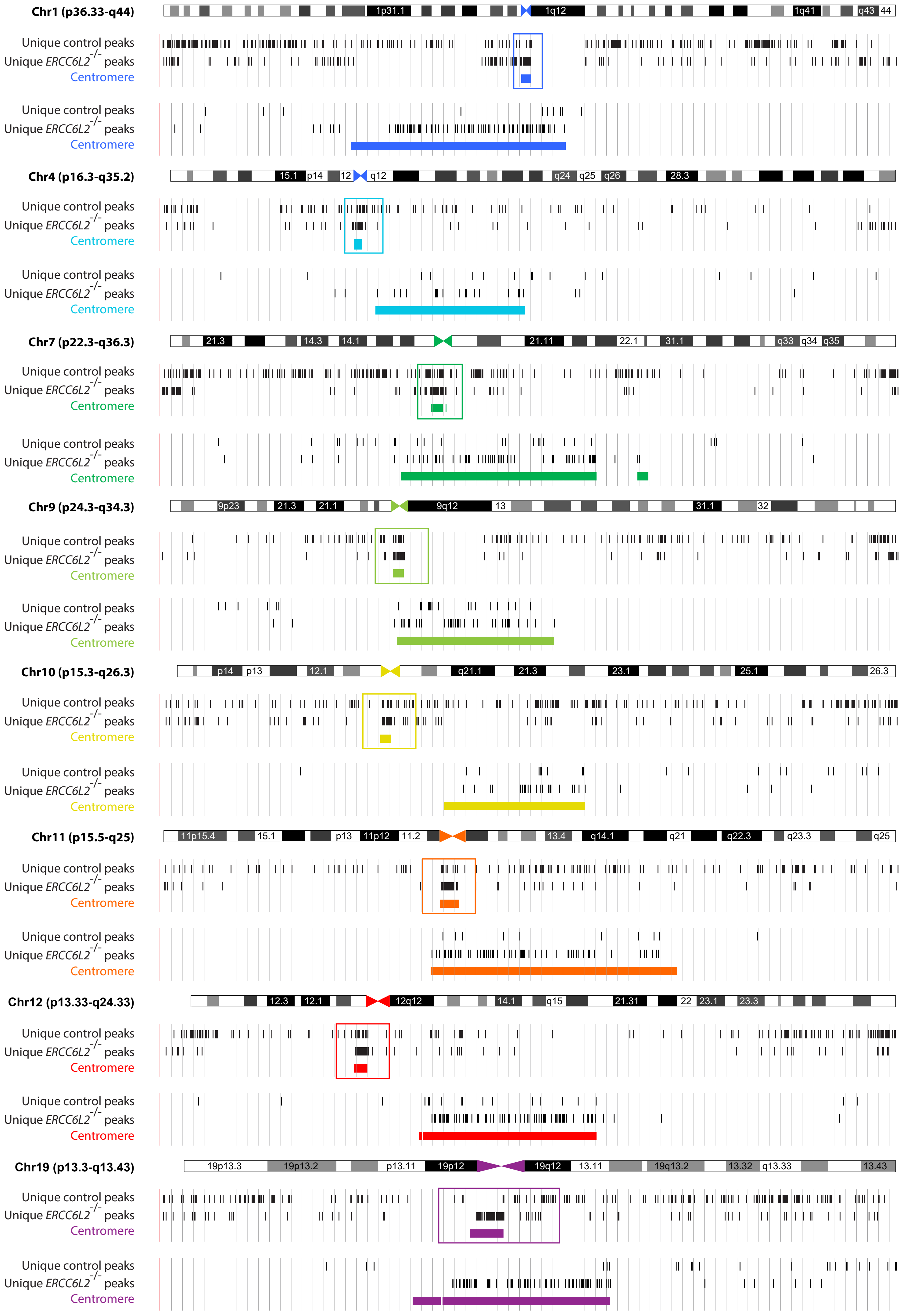
Chr1 (p36.33-q44)
1p31.1
1q12
1q41
q43
44
Unique control peaks
Unique ERCC6L2-/- peaks
Centromere
Unique control peaks
Unique ERCC6L2-/- peaks
Centromere
Chr4 (p16.3-q35.2)
15.1
p14
12
q12
q24
q25
q26
28.3
Unique control peaks
Unique ERCC6L2-/- peaks
Centromere
Unique control peaks
Unique ERCC6L2-/- peaks
Centromere
Chr7 (p22.3-q36.3)
21.3
14.3
14.1
21.11
22.1
31.1
q33
q34
q35
Unique control peaks
Unique ERCC6L2-/- peaks
Centromere
Unique control peaks
Unique ERCC6L2-/- peaks
Centromere
Chr9 (p24.3-q34.3)
9p23
21.3
21.1
9q12
13
31.1
32
Unique control peaks
Unique ERCC6L2-/- peaks
Centromere
Unique control peaks
Unique ERCC6L2-/- peaks
Centromere
Chr10 (p15.3-q26.3)
p14
p13
12.1
q21.1
21.3
23.1
25.1
26.3
Unique control peaks
Unique ERCC6L2-/- peaks
Centromere
Unique control peaks
Unique ERCC6L2-/- peaks
Centromere
Chr11 (p15.5-q25)
11p15.4
15.1
p13
11p12
11.2
13.4
q14.1
q21
q22.3
q23.3
q25
Unique control peaks
Unique ERCC6L2-/- peaks
Centromere
Unique control peaks
Unique ERCC6L2-/- peaks
Centromere
Chr12 (p13.33-q24.33)
12.3
12.1
12q12
14.1
q15
21.31
22
23.1
23.3
Unique control peaks
Unique ERCC6L2-/- peaks
Centromere
Unique control peaks
Unique ERCC6L2-/- peaks
Centromere
Chr19 (p13.3-q13.43)
19p13.3
19p13.2
p13.11
19p12
19q12
13.11
19q13.2
13.32
q13.33
13.43
Unique control peaks
Unique ERCC6L2-/- peaks
Centromere
Unique control peaks
Unique ERCC6L2-/- peaks
Centromere

Figure S3.

B

Unique Control motifs

Motif	Complement	Reverse complement	P-value	Sites
1	GCTCACTGCAACCT	AGGTTGCAGTGAGC	$1.4e^{-014}$	478/3771
2	CTCCCTCCCTCCCT	AGGGAGGGAGGGAG	$2.0e^{-014}$	637/3771
3	ARAGAAAGAAAGAAA	TTTCTTTCTTTCTYT	$1.1e^{-013}$	552/3771
4	CCTCAGCCTCCCRAG	CTYGGGAGGCTGAGG	$7.3e^{-012}$	410/3771
5	TGGGYRACAGAGY	RCTCTGTYRCCCA	$2.9e^{-011}$	407/3771
6	GAAGGAAGGAAGGAA	TTCCTTCCTTCCTTC	$3.8e^{-011}$	592/3771
7	GGCTGGAGTGCAGT	ACTGCACTCCAGCC	$5.9e^{-011}$	547/3771
8	AGAGAGAGAGAAAGA	TCTTTCTCTCTCTCT	$1.7e^{-010}$	720/3771
9	CTGTAATCCCAGCT	AGCTGGGATTACAG	$9.7e^{-009}$	414/3771
10	ATYGCTTGAACCC	GGGTTCAAGCRAT	$1.6e^{-008}$	447/3771

Simple repeats

Unique *ERCC6L2*$^{-/-}$ motifs

Motif	Complement	Reverse complement	P-value	Sites
1	CCCGTTTCCAACGAA	TTCGTTGGAAACGGG	$4.1e^{-018}$	590/1614
2	AAATATCCACTTGCA	TGCAAGTGGATATTT	$8.1e^{-018}$	589/1614
3	AACACTCTKTTTGTA	TACAAAMAGAGTGTT	$8.1e^{-018}$	580/1614
4	ACATCACAAAGDAGT	ACTHCTTTGTGATGT	$8.1e^{-018}$	580/1614
5	CTAGACAGAAKCATT	AATGMTTCTGTCTAG	$1.6e^{-017}$	586/1614
6	AAGGAAATATCTTCS	WGAAGATATTTCCTT	$1.6e^{-017}$	578/1614
7	AWCTGCTCTGTCWAA	TTSGACAGAGCAGST	$1.6e^{-017}$	573/1614
8	GCTTTGAGGYCWATG	CATSGRCCTCAAAGC	$6.5e^{-017}$	565/1614
9	ACAGAGTTGAACCTT	AAGGTTCAACTCTGT	$2.4e^{-016}$	587/1614
10	TGCATTCAASTC	GAWTTGAATGCA	$2.6e^{-014}$	571/1614

Centromeric repeats

Figure S3. Nascent DNA sequencing reveals differences in DNA replication in control and ERCC6L2$^{-/-}$ cells. Related to Figure 3.
(A) Comparison of the unique control and ERCC6L2$^{-/-}$ peaks on selected chromosomes identified by nascent DNA sequencing. Notable differences are observed in centromeric regions, shown as zoomed views of the framed 10 Mb regions. (B) List of top 10 recurring motifs among unique control and ERCC6L2$^{-/-}$ peaks identified by the MEME suite[1]. Shown are P values and numbers of sites. In addition to standard symbols, MEME uses ambiguous symbols R (purine), Y (pyrimidine), M (A, C), K (G, T), S (C,G), and W (A,T).

Figure S4.

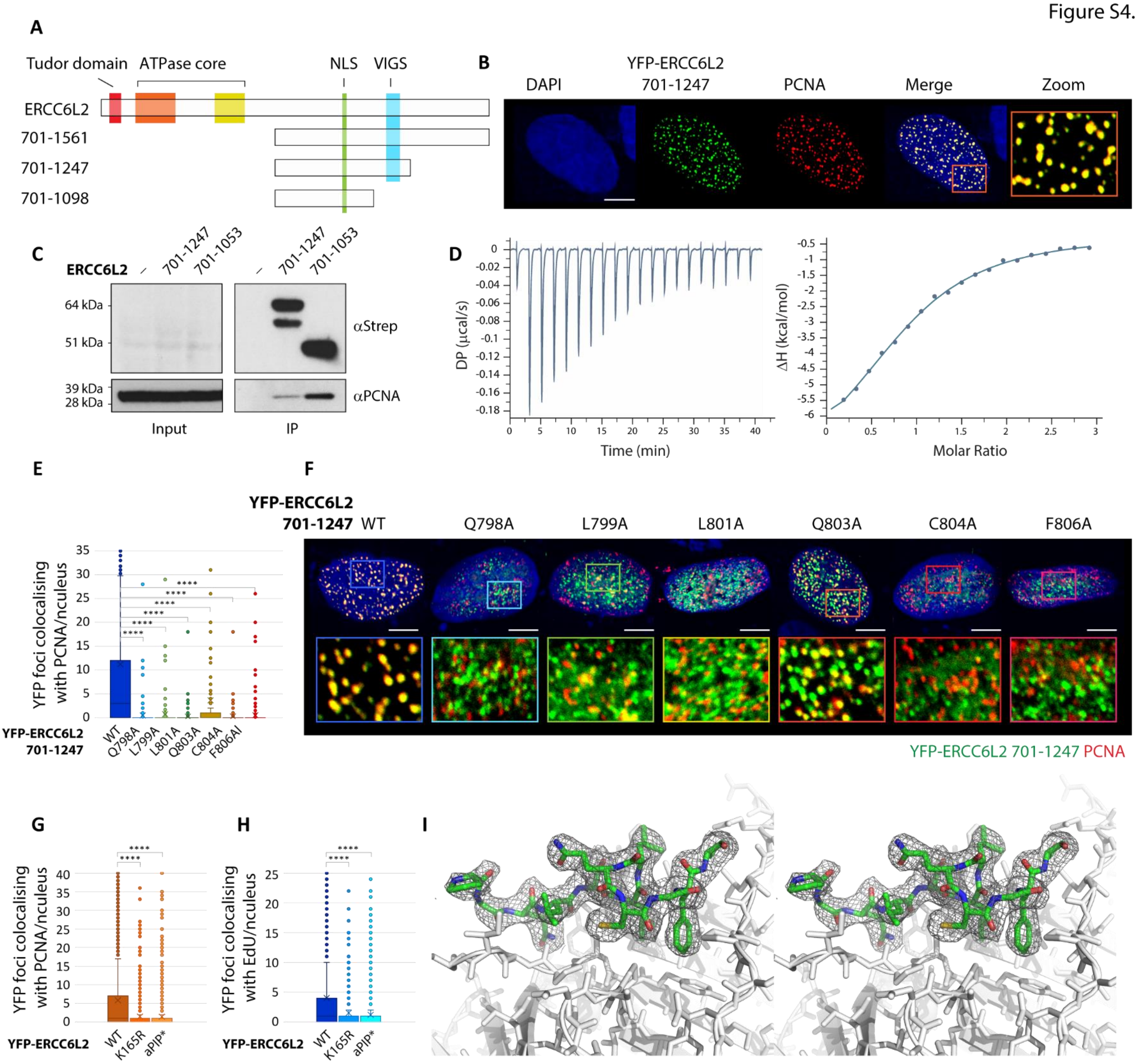

Figure S4. Identification of an atypical PCNA-binding motif in ERCC6L2 protein. Related to Figure 4.

(A) Schematic representation of different ERCC6L2 fragments used in co-localisation studies. (B) Colocalisation of the C-terminal ERCC6L2$^{701-1247}$ fragment with endogenous PCNA. (C) Interaction of the C-terminal ERCC6L2 fragments with endogenous PCNA. 293T cells were transiently transfected with the indicated Twin-Strep constructs and subjected to immunoprecipitation on StrepTactin beads. Immunocomplexes were eluted with biotin and immunoblotted with the indicated antibodies. (D) Isothermal titration calorimetry measurement with the ERCC6L2 aPIP-box peptide. Shown are thermograms and the binding isotherms from the integrated thermogram fits, with the one-site model (as analysed by MicroCal PEAQ-ITC Analysis Software). (E) Comparison of the C-terminal ERCC6L2$^{701-1247}$ wild type and mutant fragments' ability to form foci, and to co-localise with PCNA. N(cells)>200. (F) Expression of the wild type and mutant YFP-ERCC6L2$^{701-1247}$ constructs in U2OS cells stained against endogenous PCNA. Magnified images of selected foci (in frames) are shown below. Quantification of colocalisation is shown in Figure 4H. (G) Colocalisation of wild type YFP-ERCC6L2, ATPase dead ERCC6L2 K165R and ERCC6L2 aPIP* mutant (Q798A, C804A, F806A) with PCNA. N(cells)>1700. (H) Colocalisation of wild type YFP-ERCC6L2, ATPase dead ERCC6L2 K165R and ERCC6L2 aPIP* mutant (Q798A, C804A, F806A) with) with EdU. Transfected cells were incubated with 10 mM EdU for 20 min prior fixation and EdU staining. N(cells)>850. (I) Stereo view of the ERCC6L2 aPIP-box peptide electron density map. 2Fo−Fc density map is contoured at 1.0 σ and coloured grey. ERCC6L2 aPIP-box peptide is shown as green and PCNA as white sticks. E, G and H: Statistics calculated by t-Test assuming unequal variances; ****P ≤ 0.0001. B, F: Scale bar: 5 μm.

Figure S5.

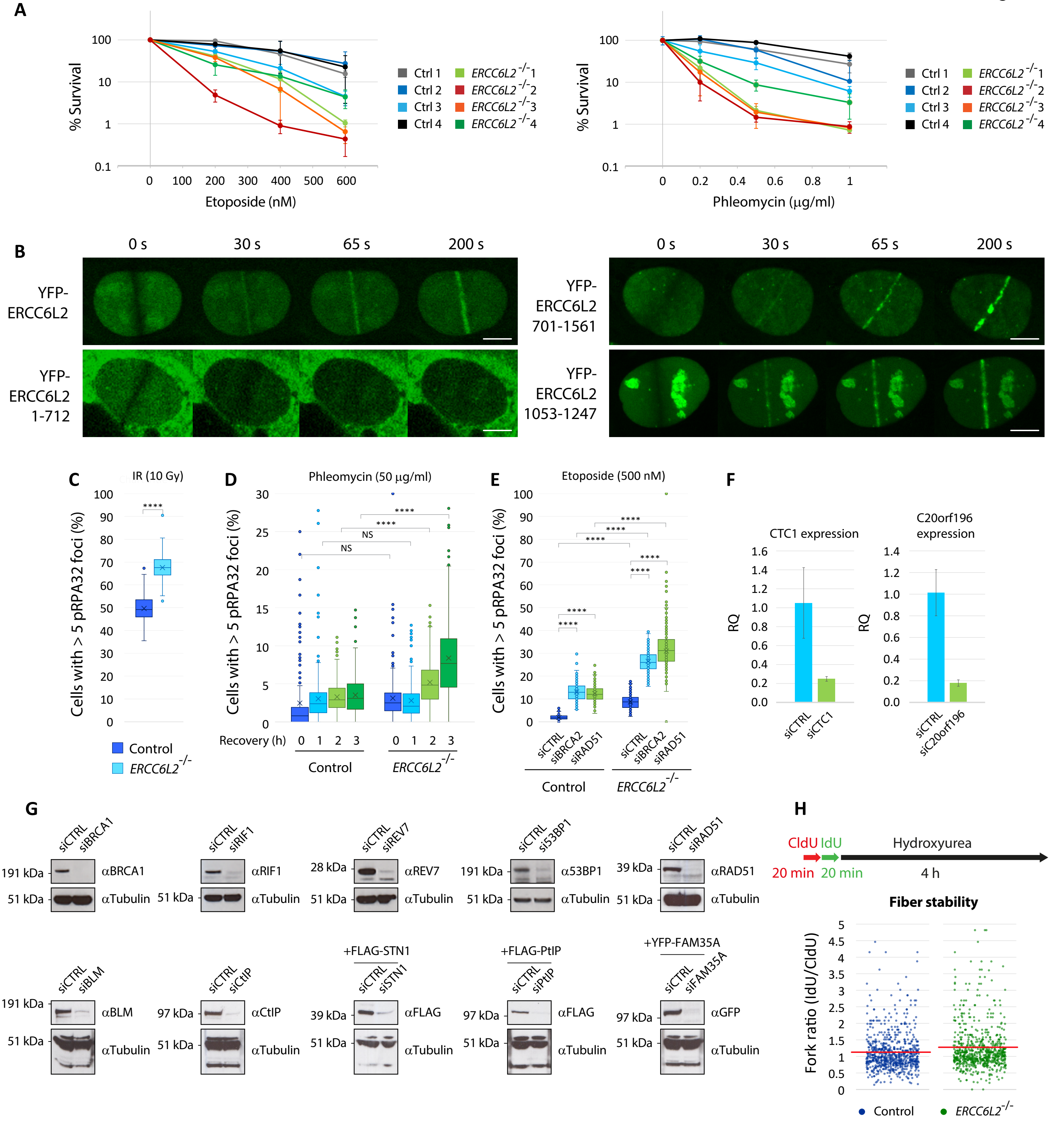

Figure S5. ERCC6L2 deficiency causes sensitivity to genotoxic stress. Related to Figure 5.
(A) Clonogenic survival of control and ERCC6L2$^{-/-}$ U2OS cells exposed to etoposide and phleomycin. Error bars represent standard errors from 3 independent experiments. (B) ERCC6L2 is recruited to sites of DNA damage through its C-terminal domain. U2OS cells were transfected with YFP-ERCC6L2 constructs and monitored by live cell imaging following laser induced DNA damage. Scale bar: 5 µm. (C) Quantification of ssDNA accumulation in control and ERCC6L2$^{-/-}$ U2OS cells. Cells were irradiated with 10 Gy and allowed to recover for 6 h. pRPA staining was used as a surrogate marker for ssDNA. (D) Kinetics of the ssDNA accumulation in control and ERCC6L2$^{-/-}$ U2OS cells. Cells were treated with 50 µg/ml phleomycin for 1 h, and allowed to recover in normal media for the indicated length of time. (E) Downregulation of BRCA2 or RAD51 exacerbates pRPA accumulation in and ERCC6L2$^{-/-}$ cells. Cells were transfected with the indicated siRNAs and treated with 500nM etoposide for 6h. (F) Validation of siRNA against CTC1 and C20orf196. Cells were transfected with the siRNAs on 2 consecutive days and collected 48 hours after first transfection. Expression was quantified by qPCR from triplicates. Bars represent means with standard deviations. (G) Validation of the indicated siRNAs by Western blot. For BRCA1, RIF1, REV7, 53BP1, BLM and CtIP, cells were transfected as in F, and endogenous proteins were detected by specific antibodies. For STN1, PtIP and FAM35A, cells were transfected with the indicated FLAG- or YFP- constructs 6 hours after first siRNA transfection, and collected 24 hours after second siRNA transfection. Expression was detected by anti-FLAG or anti-GFP antibodies, as indicated. (H) Quantification of nascent DNA degradation calculated from the ratio of CldU and IdU labelled tracks. Following sequential incubation with CldU and IdU analogues, cells were inbubated with 3 mM hydroxyurea for 4 h to induce nascent DNA degradation. N(tracks)>700. C-E: Statistics calculated by t-Test assuming unequal variances; *P ≤ 0.05, **P ≤ 0.01, ***P ≤ 0.001, ****P ≤ 0.0001, NS not significant. C-E. N(images)>250, N(cells)>30,000.

Figure S6.

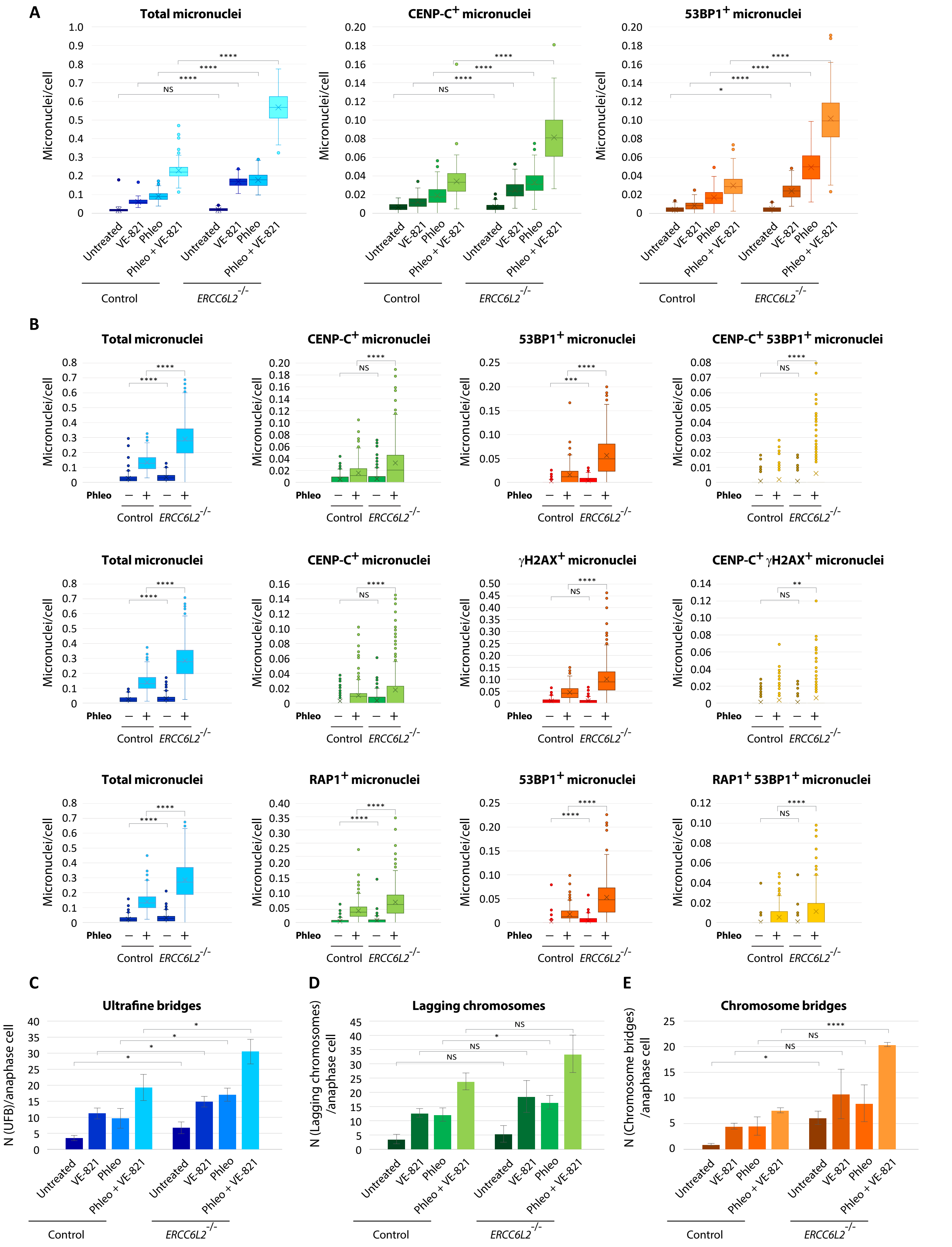
A
Total micronuclei
CENP-C+ micronuclei
53BP1+ micronuclei
Micronuclei/cell
Untreated
VE-821
Phleo
Phleo + VE-821
Control
ERCC6L2-/-
B
Total micronuclei
CENP-C+ micronuclei
53BP1+ micronuclei
CENP-C+ 53BP1+ micronuclei
γH2AX+ micronuclei
CENP-C+ γH2AX+ micronuclei
RAP1+ micronuclei
RAP1+ 53BP1+ micronuclei
Phleo
C
Ultrafine bridges
N (UFB)/anaphase cell
D
Lagging chromosomes
N (Lagging chromosomes) /anaphase cell
E
Chromosome bridges
N (Chromosome bridges) /anaphase cell

Figure S6. ERCC6L2 deficiency causes nuclear abnormalities. Related to Figure 6.
(A) Quantification of micronuclei induced by phleomycin, VE-821, or a combination of phleomycin and VE-821. Cells were grown in the absence or presence of damaging agents for 2 days. CENPC and 53BP1 were used as centromeric and DSB markers, respectively. N(images) >140, N(cells)>50,000. (B) Quantification of micronuclei subtypes in control and ERCC6L2$^{-/-}$ U2OS cells, as indicated. 53BP1 or γH2AX were used as DSB markers; CENP-C was used as a centromere marker; Telomeric repeat-binding factor 2-interacting protein 1 (RAP1) was used as a telomere marker. N(images)>400, N(cells)>30,000. (C) Quantification of UFBs induced by genotoxic agents, as indicated. Conditions were as in A. N(total anaphase cells)>260. Bars represent means with standard deviations. (D) Quantification of lagging chromosomes induced by genotoxic agents, as indicated. Conditions were as in A. N(total anaphase cells)>260. Bars represent means with standard deviations. (E) Quantification of chromosome bridges induced by genotoxic agents, as indicated. Conditions were as in A. N(total anaphase cells)>260. Bars represent means with standard deviations. A-E: Statistics calculated by t-Test assuming unequal variances; *P ≤ 0.05, **P ≤ 0.01, ***P ≤ 0.001, ****P ≤ 0.0001, NS not significant.

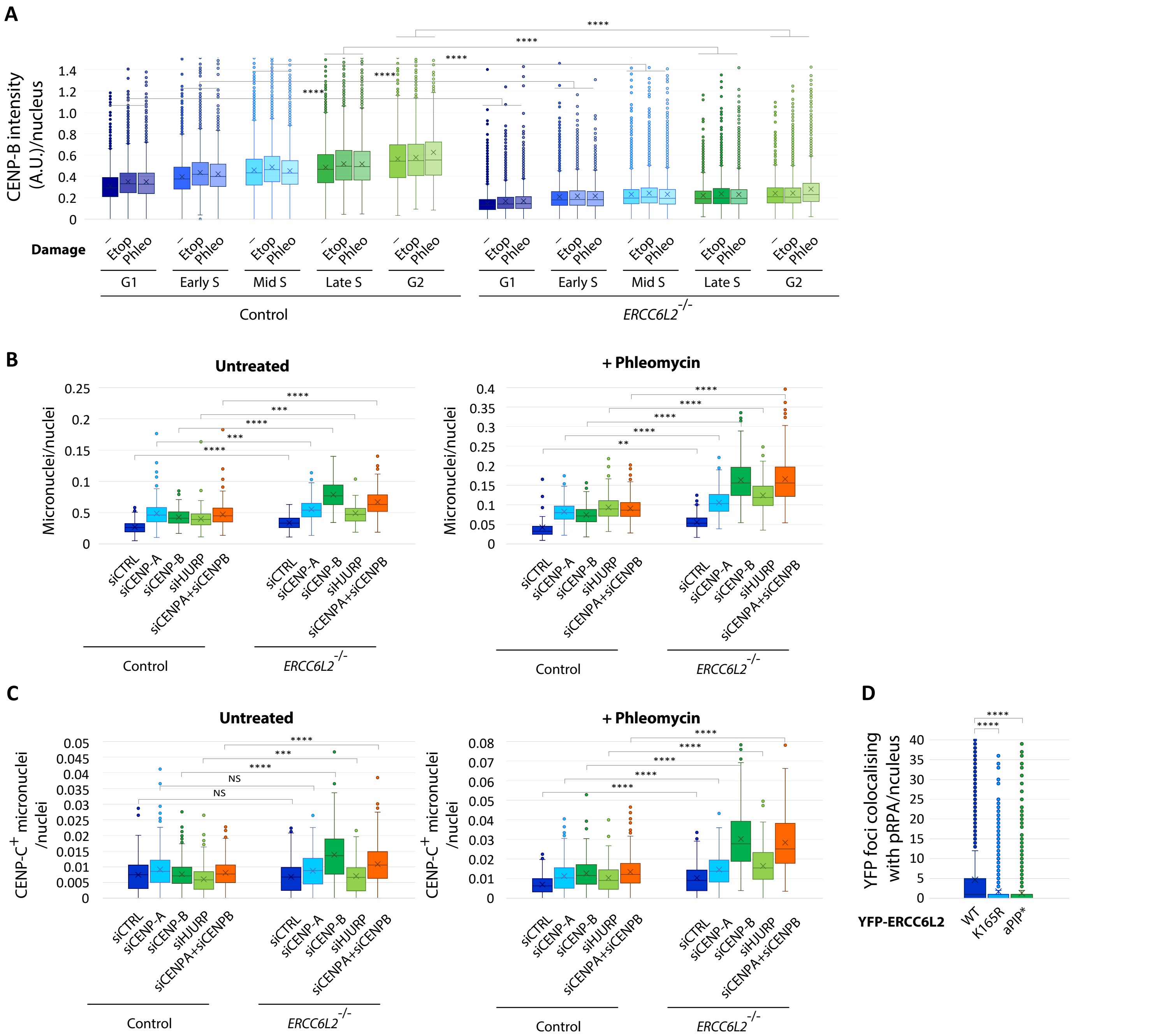

Figure S7. ERCC6L2 functions at centromeres independently of DNA damage. Related to Figure 6.
(A) CENP-B intensities are not affected by DNA damage. CENP-B intensities were measured in untreated cells, and cells exposed to genotoxic stress. Where indicated, cells were exposed to 25 μg/ml phleomycin for 1 h and allowed to recover for 5 h, or they were treated with 500 nM etoposide for 6 h. Different subpopulations were identified as in Figure S2A. N(cells)>30,000. (B) Quantification of micronuclei in cells transfected with the indicated siRNA. Cells were transfected with siRNA on two consecutive days, and either incubated in normal media, or exposed to 25 μg/ml phleomycin for another 24 h before fixation and staining. N(images)>180, N(cells)>20,000. (C) Quantification of centromere positive micronuclei in cells treated as in B. N(images)>180, N(cells)>20,000. (D) Colocalisation of YFP-ERCC6L2 wilt type, ATPase dead K165 ERCC6L2 and aPIP* mutant (Q798A, C804A, F806A) with pRPA. Transfected cells were treated with 20 μg/ml phleomycin for 1 h and allowed to recover for additional 5 h before fixation and staining. N(cells)>2100. A-D: Statistics calculated by t-Test assuming unequal variances; *P ≤ 0.05, **P ≤ 0.01, ***P ≤ 0.001, ****P ≤ 0.0001, NS not significant.

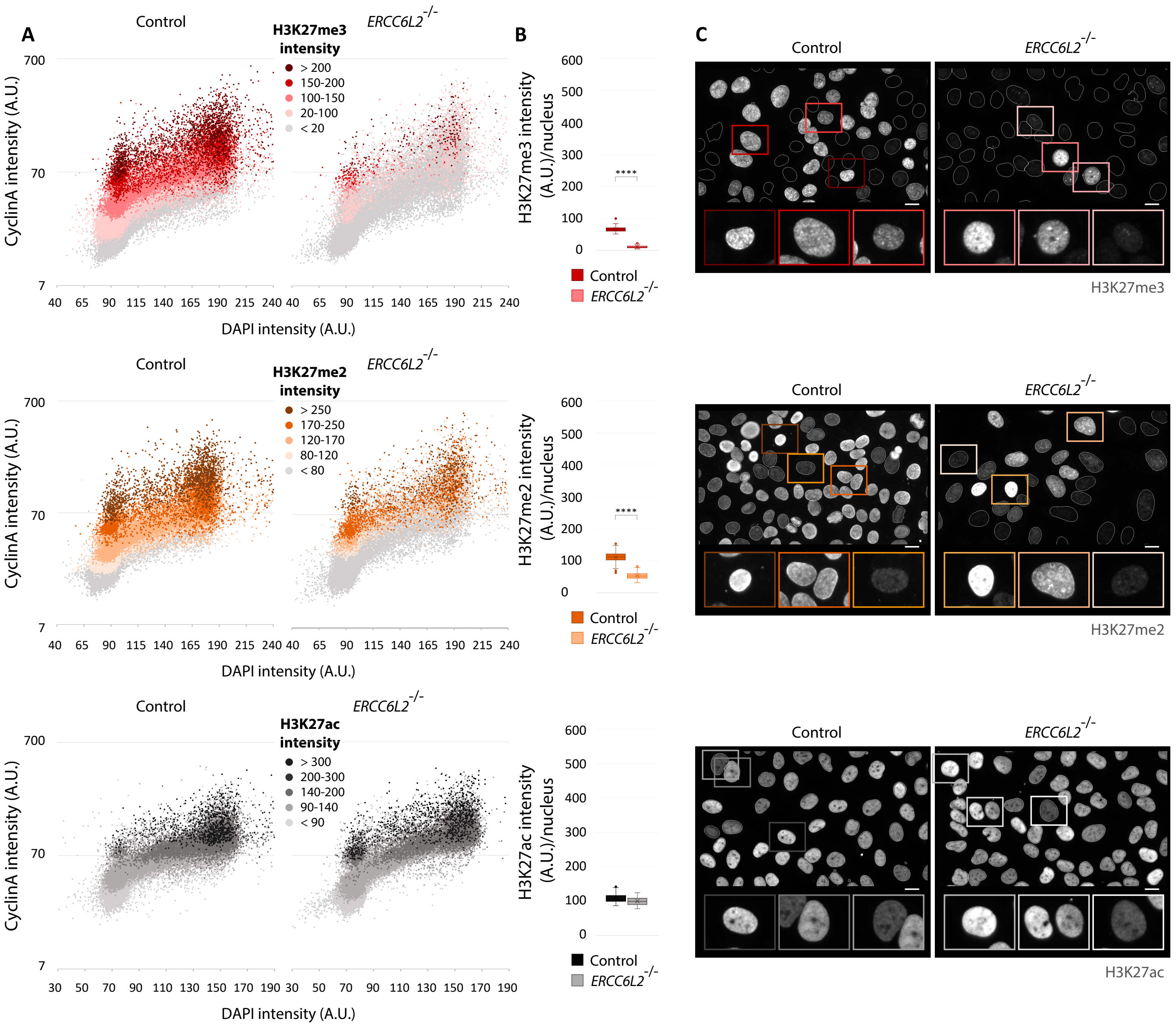

Figure S8. ERCC6L2$^{-/-}$ cells show signs of altered chromatin structure. Related to Figure 6.
(A) QIBC assays measuring H3K27me3, H3K27me2, H3K27ac intensities in control and ERCC6L2$^{-/-}$ U2OS cells. Subpopulations of cells are identified using cyclin A and DAPI intensities. Individual cells were coloured according to the relative H3K27me3, H3K27me2, H3K27ac intensities, as indicated. N(images)>180, N(cells)>35,000. (B) Box and whisker plots measuring average intensities of H3K27me3, H3K27me2, H3K27ac intensities in control and ERCC6L2$^{-/-}$ U2OS cells. N(images)>180, N(cells)>35,000. (C) Representative images used for quantifications in A. Cells were stained against H3K27me3, H3K27me2, and H3K27ac, as indicated. Shown are expressions of proteins within outlined nuclei, with zoomed images of cells in coloured frames displaying different levels of intensities. Scale bar: 10 µm.B: Statistics calculated by t-Test assuming unequal variances; ****P ≤ 0.0001.

Figure S9.

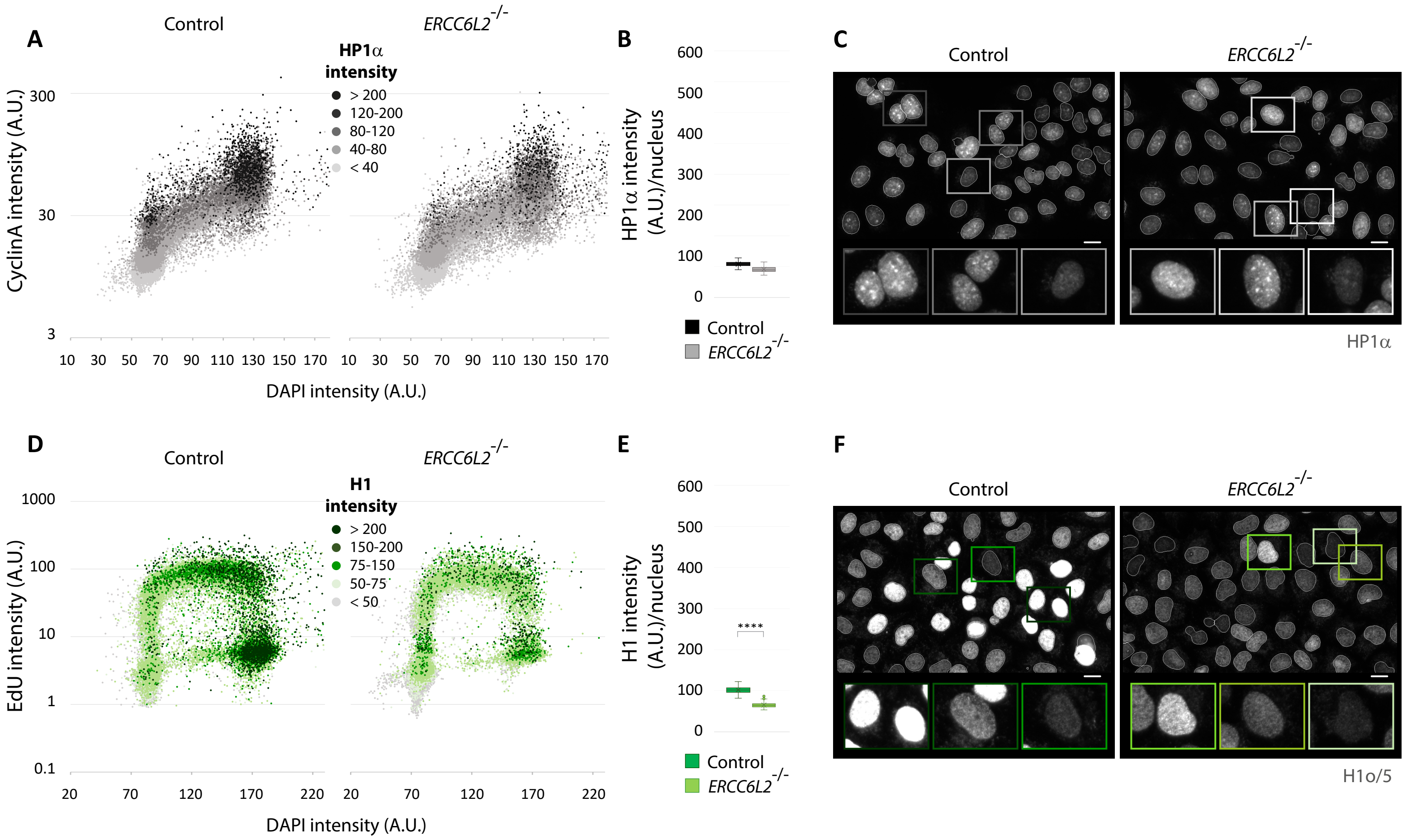

Figure S9. Comparisons of HP1α and H1 in control and ERCC6L2$^{-/-}$ cells. Related to Figure 6.
(A) QIBC assays measuring HP1α intensities in control and ERCC6L2$^{-/-}$ U2OS cells. Subpopulations of cells are identified using cyclin A and DAPI intensities. Individual cells were coloured according to the relative HP1α intensities, as indicated. N(images)>200, N(cells)>28,000. (B)Box and whisker plots measuring average HP1α intensities in control and ERCC6L2$^{-/-}$ U2OS cells. N(images)>200, N(cells)>28,000. (C) Representative images used for quantifications in A. Cells were stained against HP1α. Shown are expressions of HP1α within outlined nuclei, with zoomed images of cells in coloured frames displaying different levels of intensities. (D) QIBC assays measuring H1 intensities in control and ERCC6L2$^{-/-}$ U2OS cells. Subpopulations of cells are identified using EdU and DAPI intensities. Individual cells were coloured according to the relative H1 intensities, as indicated. N(images)>200, N(cells)>33,000. (E) Box and whisker plots measuring average H1 intensities in control and ERCC6L2$^{-/-}$ U2OS cells. N(images)>200, N(cells)>33,000. (F) Representative images used for quantifications in D. Cells were stained using H1o/H5 antibody. Shown are expressions of H1 within outlined nuclei, with zoomed images of cells in coloured frames displaying different levels of intensities. C, F: Scale bar: 10 μm. E: Statistics calculated by t-Test assuming unequal variances; ****P ≤ 0.0001.

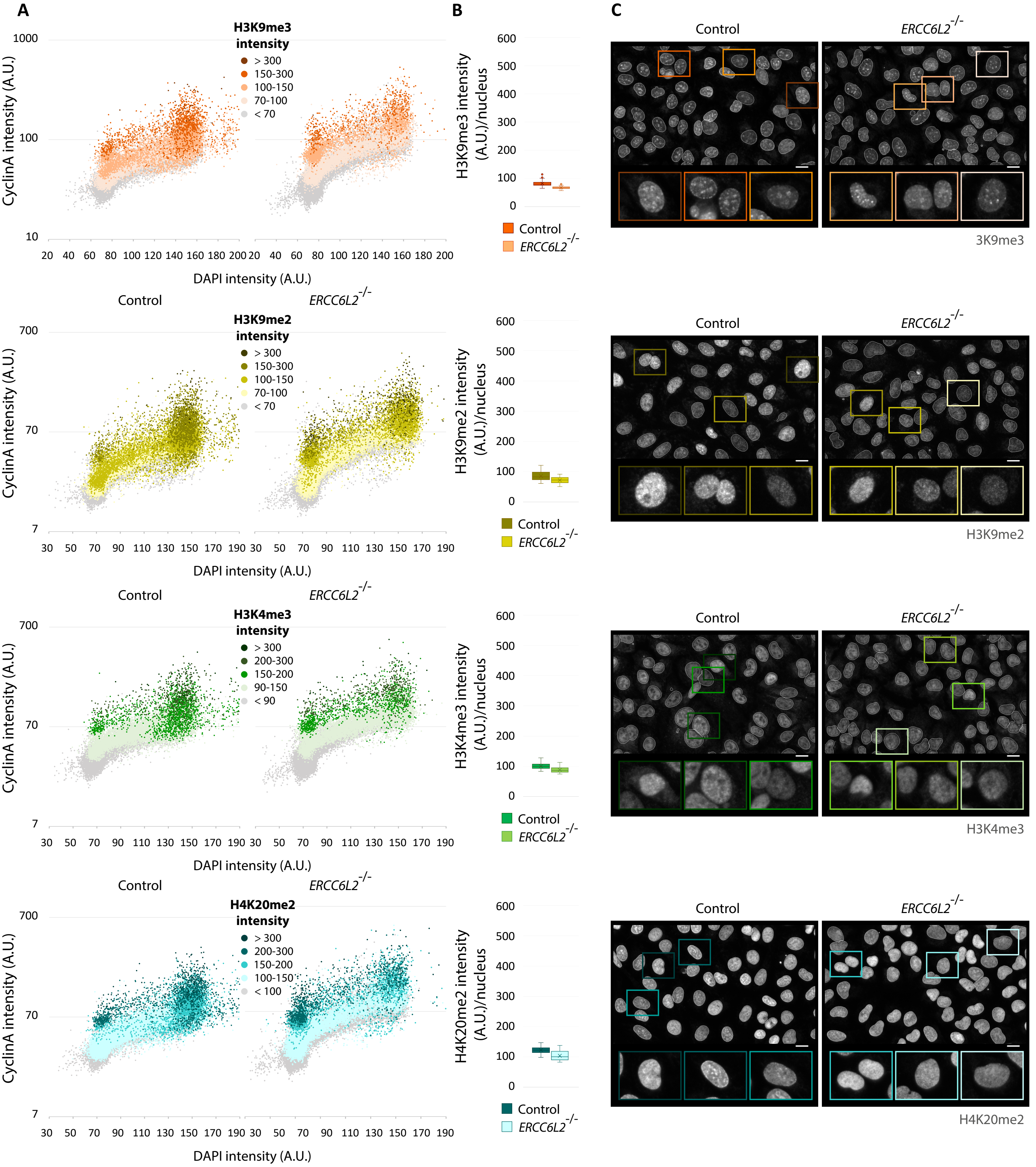

Figure S10. ERCC6L2 deficiency does not significantly affect some chromatin marks. Related to Figure 6.
(A) QIBC assays measuring H3K9me3, H3K9me2, H3K4me3 and H4K20me2 intensities in control and ERCC6L2$^{-/-}$ U2OS cells. Subpopulations of cells are identified using cyclin A and DAPI intensities. Individual cells were coloured according to the relative intensities, as indicated. N(images)>180, N(cells)>30,000. (B) Box and whisker plots measuring average H3K9me3, H3K9me2, H3K4me3 and H4K20me2 intensities in control and ERCC6L2$^{-/-}$ U2OS cells cells. N(images)>180, N(cells)>30,000. (C) Representative images used for quantifications in A. Cells were stained against H3K9me3, H3K9me2, H3K4me3 and H4K20me2, as indicated. Shown are expressions of proteins within outlined nuclei, with zoomed images of cells in coloured frames displaying different levels of intensities. Scale bar: 10 μm.

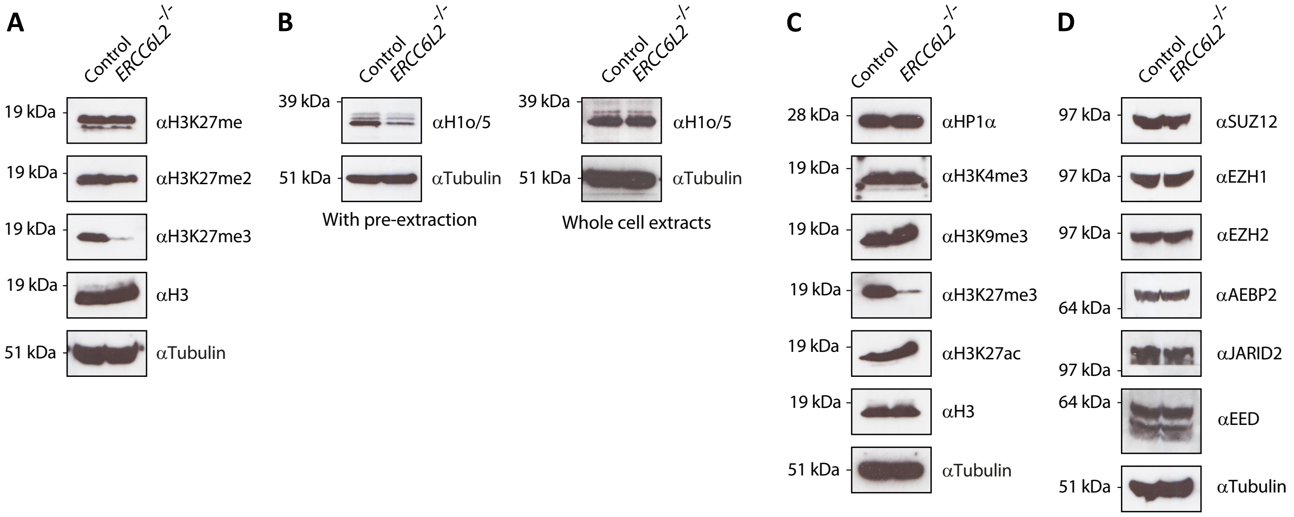

Figure S11. Comparisons of chromatin marks in control and ERCC6L2$^{-/-}$ cells by Western blot. Related to Figure 6.
(A) Analysis of H3K27me3, H3K27me2 and H3K27me levels by western blot. Blot against histone H3 is used as a loading control. (B) Western blot analysis of H1 levels in whole cell extracts and extracts derived from cells subjected to pre-extraction with 1 % Triton X in PBS. (C) Western blot analysis of the indicated chromatin markers in control and ERCC6L2$^{-/-}$ U2OS cells. (D) ERCC6L2 deficiency does not cause downregulation of the Polycomb repressive complex 2 (PRC2), responsible for H3K27 methylation. Levels of the individual PRDC2 subunits are detected by specific antibodies.

Figure S12.

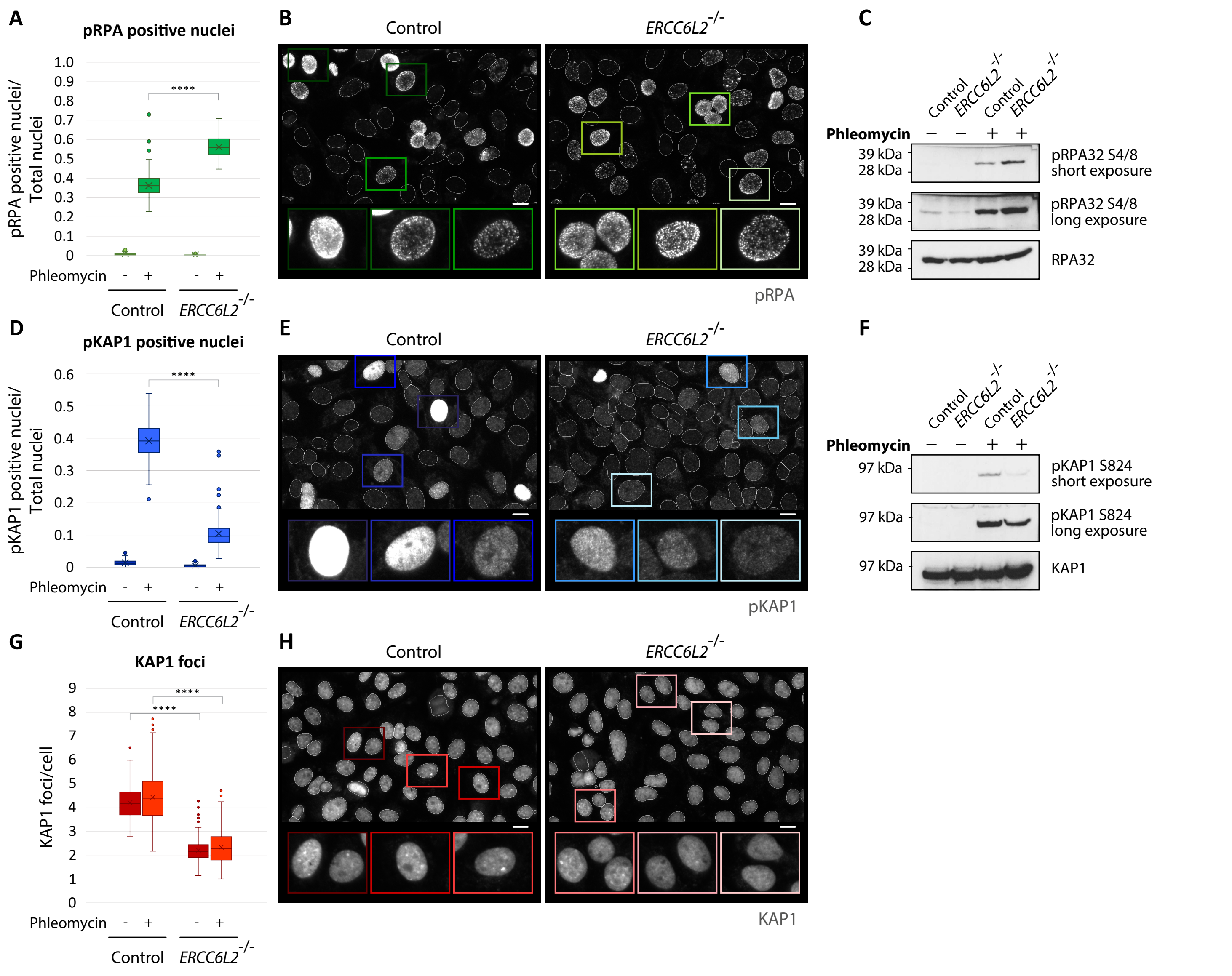

Figure S12. DNA damage-induced changes in control and ERCC6L2$^{-/-}$ cells. Related to Figure 6.
(A) Box and whisker plots measuring average pRPA intensities in control and ERCC6L2$^{-/-}$ cells. Cells were either untreated, or exposed to 50 µg/ml phleomycin for 12 h, before fixation and staining. N(images)>180, N(cells)>28,000. (B) Representative images of phleomycin treated cells used for quantifications in A. Shown are expressions of indicated proteins, with zoomed images of cells in coloured frames. (C) Analysis of pRPA levels by western blot. Treatments were as in A. (D) Box and whisker plots measuring average pKAP1 intensities in control and ERCC6L2$^{-/-}$ cells. Cells were either untreated, or exposed to 50 µg/ml phleomycin for 12 h, before fixation and staining. N(images)>180, N(cells)>28,000. (E) Representative images of phleomycin treated cells used for quantifications in D. Shown are expressions of indicated proteins, with zoomed images of cells in coloured frames. (F) Analysis of pKAP1 levels by western blot. Treatments were as in D. (G) Box and whisker plots measuring KAP1 foci in control and ERCC6L2$^{-/-}$ cells. Cells were either untreated, or exposed to 50 µg/ml phleomycin for 12 h, before fixation and staining. N(images)>180, N(cells)>28,000. B, E, H: Scale bar: 10 µm. A, D, G: Statistics calculated by t-Test assuming unequal variances; ****P ≤ 0.0001.

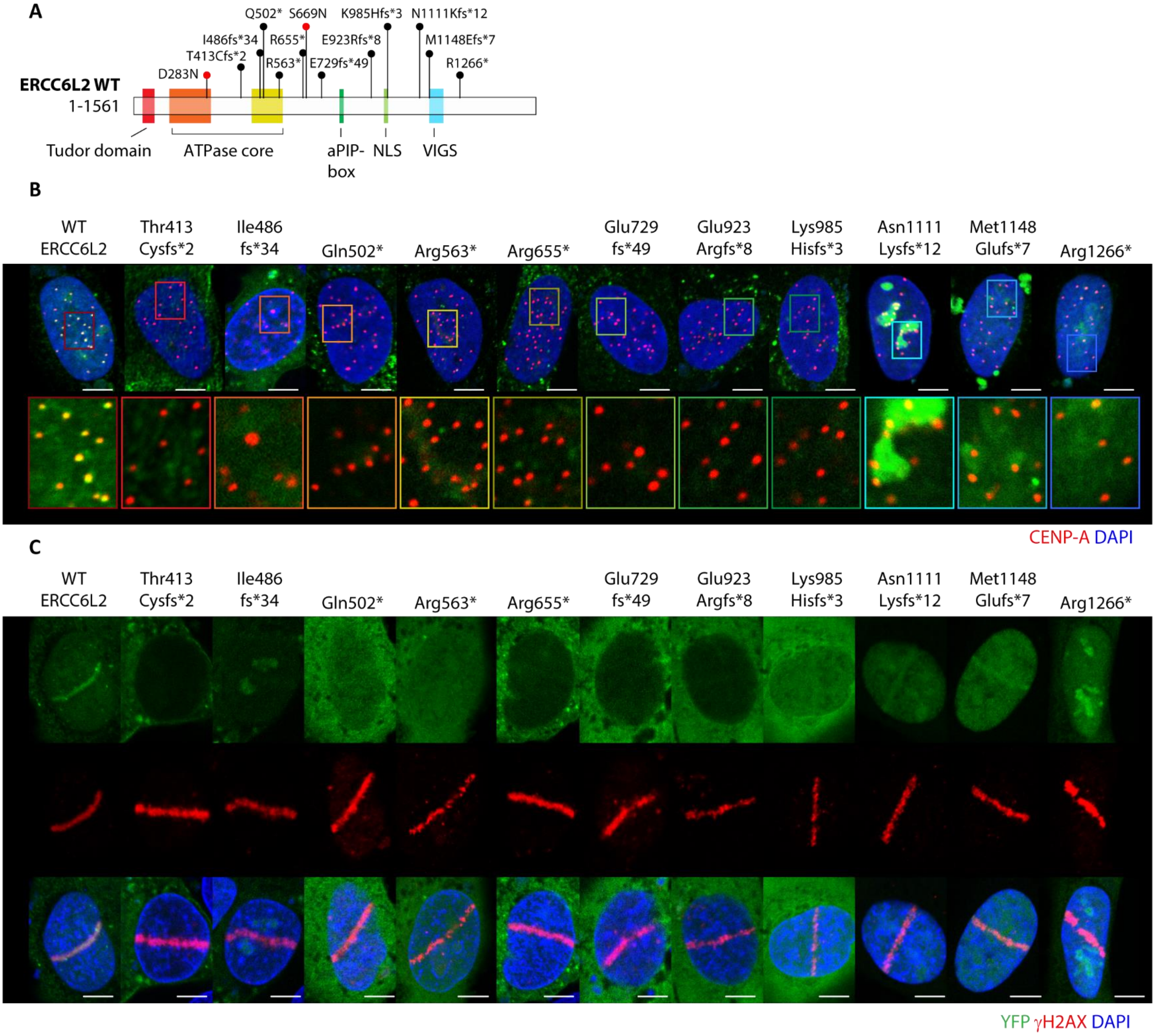

Figure S13. Pathological ERCC6L2 variants display functional deficiencies. Related to Figures 1 and 5.
(A) Schematic representation of ERCC6L2 mutations associated with IBMFS. Missense and truncating mutations are indicated as red and black dots, respectively. (B) Expression of the disease-associated YFP-ERCC6L2 variants in U2OS cells stained against CENP-A. Magnified images of selected foci (in frames) are shown below. (C) Recruitment of ERCC6L2 variants to sites of DNA damage. U2OS cells were transfected with YFP-ERCC6L2 constructs and damaged using laser microirradiation. To verify induction of DSBs, cells were immunostained with the gH2AX antibody. B, C: Scale bar: 10 mm.
